# Rapid customization system for 3D-printed splint using programmable modeling technique – a practical approach

**DOI:** 10.1186/s41205-018-0027-6

**Published:** 2018-05-25

**Authors:** Jianyou Li, Hiroya Tanaka

**Affiliations:** Graduate School of Governance and Media, Keio University, 5322 Endo, Fujisawa-shi, Kanagawa 252-0882 Japan

**Keywords:** 3D-printing, Splint, Programmable modeling, Fracture immobilization, Customization

## Abstract

**Background:**

Traditional splinting processes are skill dependent and irreversible, and patient satisfaction levels during rehabilitation are invariably lowered by the heavy structure and poor ventilation of splints. To overcome this drawback, use of the 3D-printing technology has been proposed in recent years, and there has been an increase in public awareness. However, application of 3D-printing technologies is limited by the low CAD proficiency of clinicians as well as unforeseen scan flaws within anatomic models.

A programmable modeling tool has been employed to develop a semi-automatic design system for generating a printable splint model. The modeling process was divided into five stages, and detailed steps involved in construction of the proposed system as well as automatic thickness calculation, the lattice structure, and assembly method have been thoroughly described. The proposed approach allows clinicians to verify the state of the splint model at every stage, thereby facilitating adjustment of input content and/or other parameters to help solve possible modeling issues. A finite element analysis simulation was performed to evaluate the structural strength of generated models. A fit investigation was applied on fabricated splints and volunteers to assess the wearing experience.

**Results:**

Manual modeling steps involved in complex splint designs have been programed into the proposed automatic system. Clinicians define the splinting region by drawing two curves, thereby obtaining the final model within minutes. The proposed system is capable of automatically patching up minor flaws within the limb model as well as calculating the thickness and lattice density of various splints. Large splints could be divided into three parts for simultaneous multiple printing.

**Conclusions:**

This study highlights the advantages, limitations, and possible strategies concerning application of programmable modeling tools in clinical processes, thereby aiding clinicians with lower CAD proficiencies to become adept with splint design process, thus improving the overall design efficiency of 3D-printed splints.

## Background

Upper-limb splints are employed in the treatment of immobilizing fractures, congenital deformities, and chronically degenerating orthopedic conditions. Plaster and thermoplastic sheets are primary materials employed in conventional fracture immobilization treatments. During the splinting process, the splint-fitting effect is greatly dependent on the skill and experience of the clinician because of the irreversibility of these materials and body-based contact models. Consequently, patient satisfaction levels during treatment also significantly vary depending on the clinician’s skill when performing splinting [1–3]. Inexperienced fitters may cause more pain or lead to poor immobilization. In addition, conventional splints are bulky and unsightly, thereby causing an obvious inconvenience to patients during treatment. Maintaining splints clean and dry is difficult: hence, the risk of infection spread also increases [3, 4].

In recent years, the introduction of 3D-printing techniques in orthopedic and rehabilitation practices has been extensively discussed because the use of such techniques renders it possible to customize orthoses as well as enhance patient treatment satisfaction levels [3–6]. Varieties of 3D-printed splints, which have recently been reported in media, are lightweight, well-ventilated, waterproof, and aesthetically pleasing, thereby addressing nearly all deficiencies of conventional splints [4, 6–9].

Three-dimensionally printed splints have mainly benefited from three digital techniques. Highly customized fit and comfort can be realized by means of 3D scanning [10]. Intricate lattice structural designs are possible using design computational tools. Use of 3D printing enables design of complex, individualized splints at relatively low cost [2, 4, 9, 10]. The three digitized processes involved in splint printing include [11–13].

1. acquiring splint mesh model from the patient’s affected limb surface by means of a 3D scanner.
2. designing the splint model using computer-aided design (CAD) software tools and exporting fabrication data.
3. fabricating a physical splint by using of a 3D printing device.

Nonetheless, several issues exist in the above mentioned digitization processes. The quality and accuracy of the scan of the patient’s affected limb plays a critical role in determining the success rate of the split model subsequently designed. Occurrence of irregular holes in the scan are a common sight on the dark side of the limb model and skin wrinkles are observed between fingers where the scanning light rays cannot reach [14]. In addition, it is difficult for an injured person to maintain the required posture the during 3D-scanning exercise, and even slight uncontrollable shaking of the patient’s limb can result in partial deformations or distortions appearing in the final scan. When employing the deformable-alignment technique [14–16], acquiring a complete result during the scanning process requires use of additional software, relevant techniques, and post-processing; this invariably involves increased investment of time and cost as well as specialized training to be provided to clinicians. Several CAD modeling approaches [3, 6, 14, 17] have been proposed for constructing splint models. The conventional splint-model construction technique involves dozens of steps, and the total time required depends on the operator’s CAD skills. Clinicians are invariably required to integrate the necessary design and medical knowledge and come up with a design feasible for use in the treatment. Actual interactions that occur between the clinician, CAD interface, and system feedback are not sufficiently clear; it is, therefore, difficult to evaluate the operational knowledge required by the clinician to eliminate errors that may occur during the design process. Although application of software-based tools for automatic generation of printable models has been claimed with regard to certain conceptual prototype designs of novel 3D-printed splints, the construction methodology employed and modeling mechanism have not yet been proposed [7, 8]. Finally, the printing stage takes approximately 10 h to complete splint fabrication [3, 14, 18]. In comparison, conventional splinting processes can be completed within 20 min; state-of-the-art 3D-printing solutions are, therefore, is still relatively time-consuming.

The proposed study describes development of a precompiled customization system to help clinicians design 3D-printed splints using a programmable modeling tool used in conjunction with a CAD software, thereby applying the modeling technique to patch-up small flaws in the anatomic model. The system has been designed to generate splint models for immobilization of distal radial/ulnar as well as carpal fractures. However, pathological/open fractures and fractures requiring internal fixation are excluded from proposed splint applications [6]. The complex modeling sequence in splint design has been integrated into the automatic system, and the clinician does not need to repeat lengthy modeling operations. Furthermore, operations remain virtually unaffected by CAD skills of the operator. In addition, the parametric environment enables automatic calculation of the thickness and lattice pattern of various splints and divides the splints into multiple components to facilitate efficient printing.

## Methods

This section presents operational guidelines to simply tasks involved at the 3D-scanning stage. Detailed steps and procedures followed in the development of the proposed automatic system for splint design through use of a programmable modeling tool, to address problems encountered during other stages, have also been discussed.

### Flaw-tolerant scanning

Following strategies were applied to address difficulties encountered during the scanning stage, at present.

1. Holes existing in the model may be repairable during the modeling process; however, it is difficult to restore deformations and distortions, caused by shaking, to their correct form. In this respect, flaw-tolerant scanning is highly beneficial in completing a scan more quickly, hence reducing the possibility of errors induced owing to uncontrollable shaking.
2. Additional post-production procedures after scanning must be avoided, and only simple clipping must be performed to remove unnecessary environmental background and/or body regions.

**Fig. 1:**
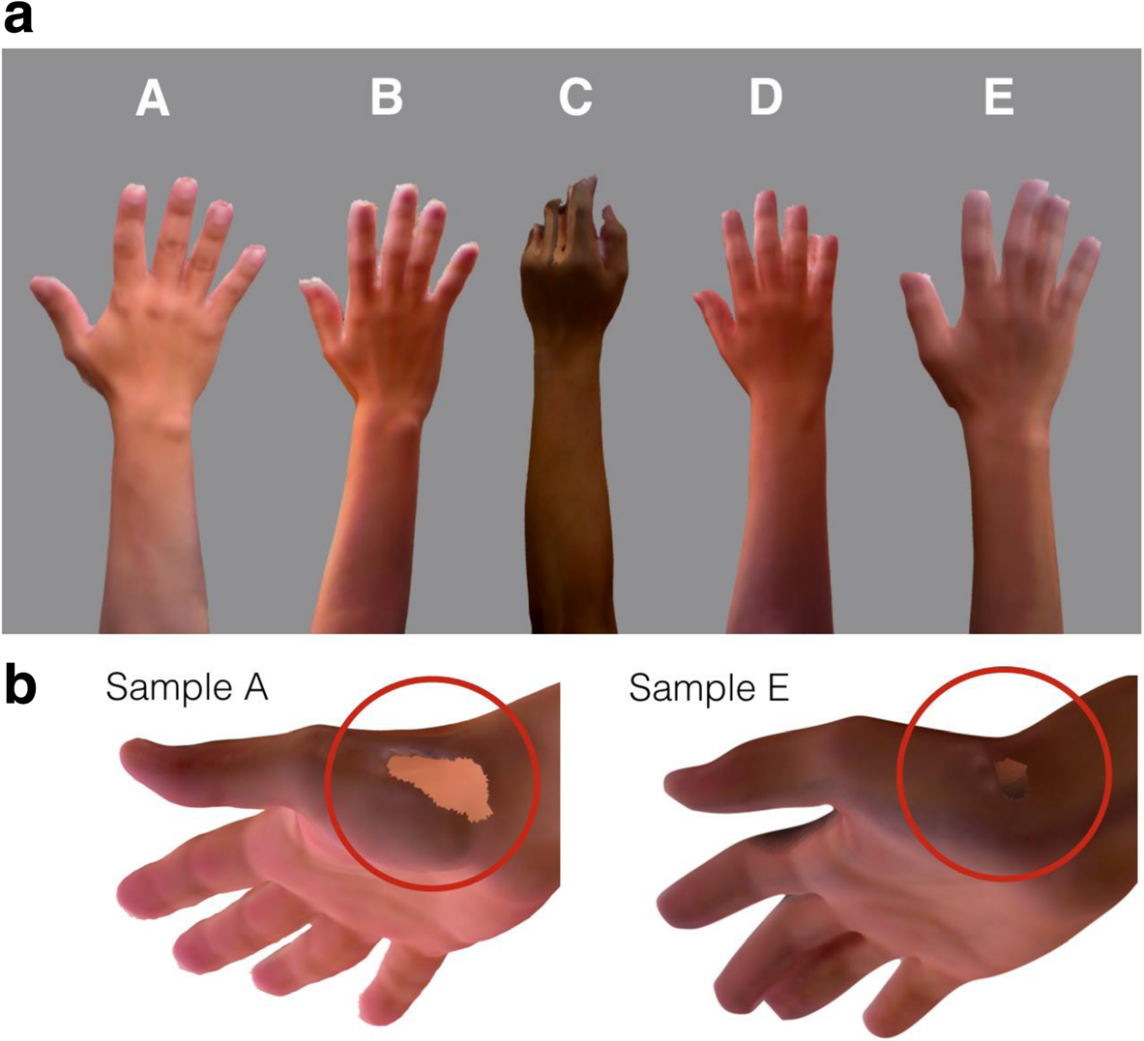
Limb samples from five volunteers (**a**) Limb samples; (**b**) Sample A and E and associated flaws

A handheld scanner, Sense (3D Systems), was used in this study for scanning and subsequently generating an output mesh model of a limb. The said scanner is affordable and lightweight, and its software offers only basic functions, such as background clipping. Five scanned samples were obtained from healthy adult volunteers (as depicted in Fig. 1(a)) whilst following above-mentioned principles. The scanned samples were used to simulate different immobilization ranges in the result, and completely scanned regions included fingers along with the palm, wrist, and fore arm. The time spent in successfully scanning these regions was in the range of 40–60 s. When performing the scanning operation, the operator must ensure plenty of elbowroom and light availability around patients’ limb. Correct use of a handheld scanner and its smooth motions along the scan path are key factors that influence efficient completion of the scanning operation. The authors recommend use of the DIY described in their published research [19]; the device can effectively reduce the total scan time to 20–30 s. No built-in light source was available within the scanner used in this study; few holes were observed to have been unintentionally created on backlight surfaces of Samples A and E (Fig. 1(b)). Although occurrence of deformations in the finger area is obvious, the palm and forearm regions remained unimpacted. The said samples were used in the subsequent modeling process.

### CAD environment, modeling goal, and program overview

#### System designer and design agent

The proposed study is not aimed at generating a tedious manual model to be employed by the clinician during treatment. The modeling task has been compartmentalized to be implemented via two roles—the system designer and design agent. An engineer or designer familiar with the use of CAD software and programming languages can follow the detailed methodology below to create an automated customization system in advance, in the capacity of a system designer. A clinician is the end user of the precompiled system, and plays the role of the design agent—to execute splint design in accordance with patients’ conditions. The clinician does not need to know how the program works, and can, therefore, instead focus on the design and evaluation of splint models.

#### Software selection

Software options for digital splint designs have been listed and comparisons between self-developed and existing CAD software have been performed in [20, 21]. Development and maintenance of self-developed software are more difficult and time-consuming. Considering programmability requirements for designing such a system, Rhinoceros 3D Version 5.0 (Robert McNeel & Associates) was used as the primary modeling environment jointly operated along with a visual programming tool—Grasshopper 3D (Robert McNeel & Associates). Rhinoceros 3D employs node-based graphics to edit and express parametric input–output relationships; it represents the primary program language employed in this study to accomplish automated modeling.

#### Splint feature definition

**Fig. 2:**
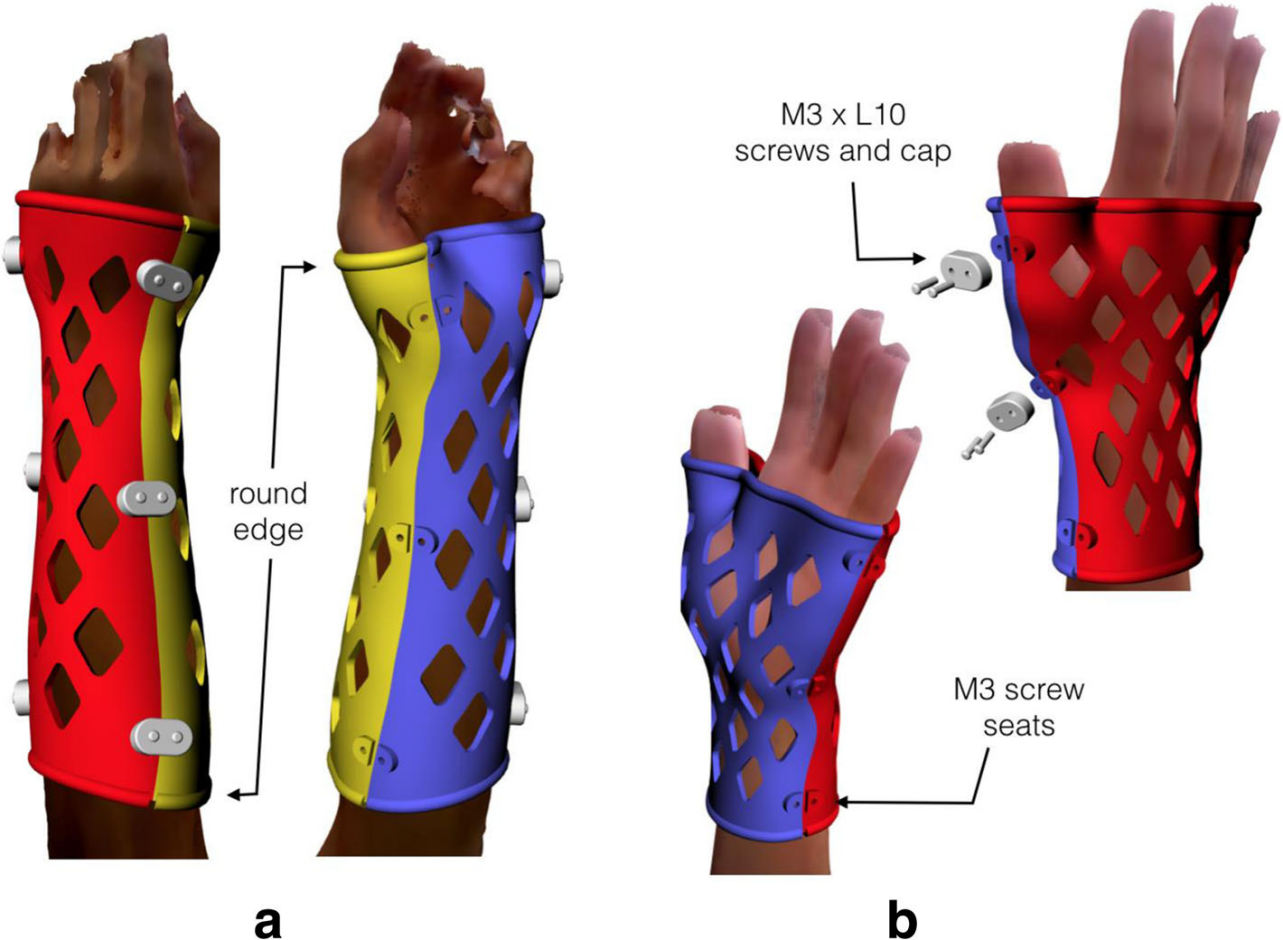
Defined splint features (**a**) 3-part set, (**b**) 2-part set for wrist splint

Splint designs generated using the proposed system exhibit features depicted in Fig. 2. Corresponding standards are as described below.

- Division: The proposed system divides the splint into a 2- or 3-part set depending on the splint size. If 2 or 3 3D printers are available for concurrent use, the splint fabrication time could be reduced to 1/2 or 1/3 the original build time, respectively.
- Lattice structure: Splint lattice patterns are created by means of a diamond structure to reduce weight and support material during printing as well as increase ventilation [20].
- Assembly method: Screw seats are generated along long edges of each divided splint part to facilitate assembly by means of plastic M3 L10 flat-point Phillips screw sets with prefabricated screw caps (Fig. 2(b)). The number of screw seats used depends on the edge length.
- Rounded edges: Splint edges are designed to be of tubular shapes to prevent skin abrasion due to sharp/rough edges [3, 6].

#### Modeling workflow and program overview

**Fig. 3:**
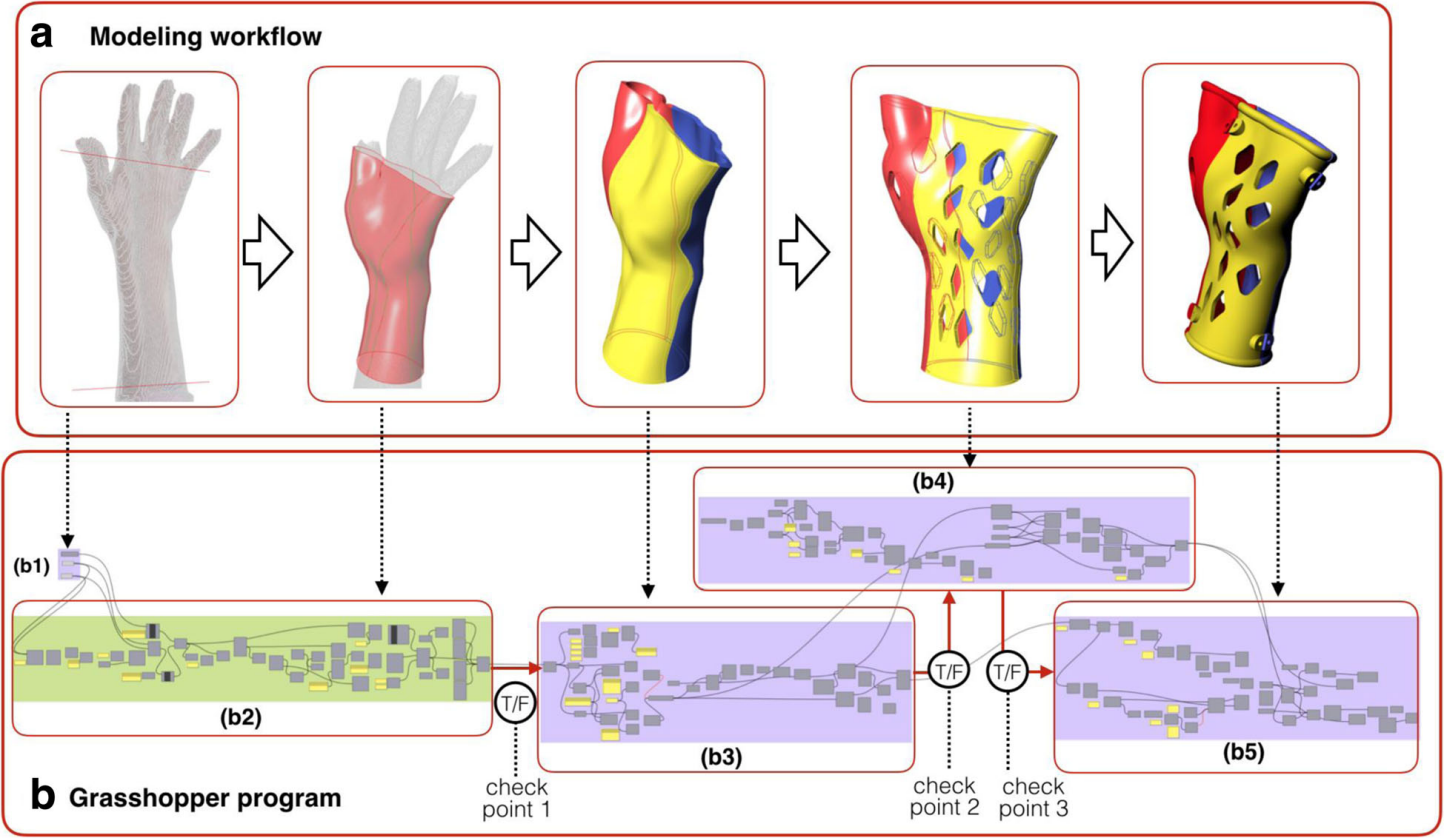
Program workflow and overview of five stages and check points. (**a**) Modeling workflow; (**b**) Grasshopper program—(b1) Input model and curves, (b2) Basic covering-surface generation, (b3) Division and thickness generation, (b4) lattice-structure creation, and (b5) Rounded-edge and screw-seat generation

In view of the above features, several different modeling sequences were tested via manual operation for building the same splint in Rhinoceros 3D, and a modeling workflow was determined after repeated comparisons, as depicted in Fig. 3(a). The modeling system was divided into five stages and switched over to Grasshopper 3D—a node-based program (Fig. 3(b)). The program comprised various components (marked by tiny gray and yellow tags) with each component performing an exclusive function, such as creating geometries, performing calculations, and making logical judgments. Individual components are connected by wires passing from left to right, thereby expressing input–output relationships. As the program is too large to be included in this manuscript, a simplified program for each stage has been described. In accordance with the workflow, program blocks in Fig. 3(b) have been marked as input model and curves (Fig. 3(b1)), basic covering-surface generation (Fig. 3(b2)), division and thickness generation (Fig. 3(b3)), lattice-structure creation (Fig. 3(b4)), and rounded-edge and screw-seat generation (Fig. 3(b5)).

All above stages are not executed in a single continuous process. Data flow during each stage is controlled by the three checkpoints depicted in Fig. 3(b). At each checkpoint, clinicians examine the splint status and confer a true value before continuing onto the next step or modifying the input area. Therefore, splint models is not generated instantaneously when the input is fulfilled; it gradually takes shape as the modeling procedure progresses. If the entire splint model were to be constructed using a single automated process, the concerned computation would be completed within tens of seconds. In that case, however, the modeling process may fail to generate a valid result if unforeseen problems are detected during modeling or computations involved in one of the stages. Furthermore, in the event of such a case, clinicians would not be able to identify the step that caused the system to fail. By using checkpoints, however, clinicians can decide to modify input curves, pattern-density parameters, and/or position of screw seats depending on the stage at which the failure occurred. Various reasons that potentially contribute to failure and corresponding appropriate responses are described in the next section. An advantage of the five-stage strategy is that it consumes a very little time to perform required computations at each stage, and the stage at which problems occur can be easily identified.

#### System programing process

An automated customization system was constructed using Grasshopper 3D as per the following procedure.

#### Limb-model import and immobilization-area assignment

**Fig. 4:**
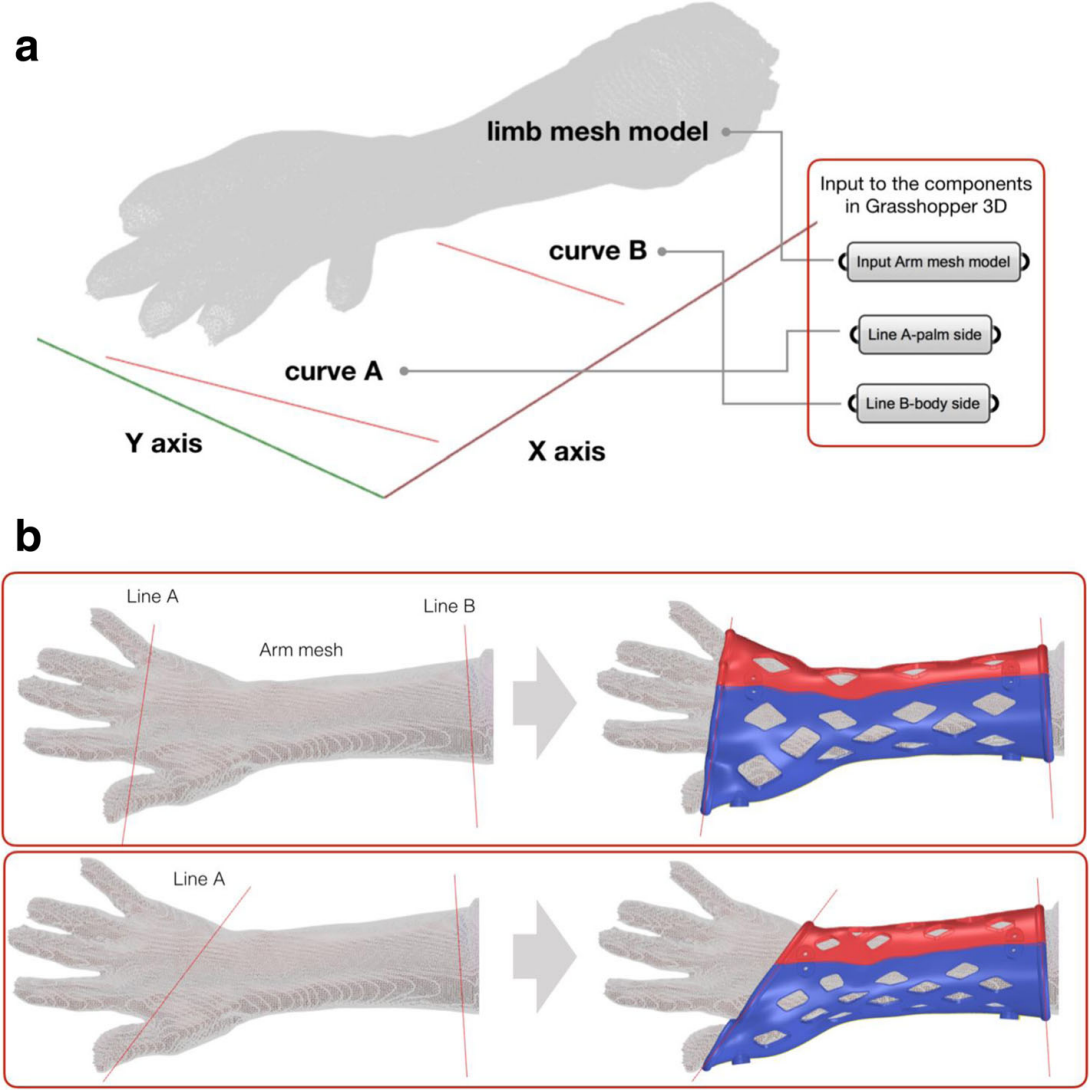
Input stage and guideline. **a** The limb mesh model is placed upon XY plane along X-axis with the palm facing up. The curves are drawn on XY plane. Select the 3 geometries and input to the parametric components in Grasshopper 3D. **b** Samples of input curves and corresponding generated splints. (**a**) Drawing input curve for defining immobile area; (**b**) Basic-surface generation—(b1) Generating gradual curves, (b2) Projected cross-sections, (b3) Covering surface, and (b4) Offsetting the covering surface; and (c1) Unclosed curves at intersection, (c2) Point extraction, and (c3) Regenerating closed curves

After import into Rhinoceros 3D, the scanned limb model must be manually placed along X-axis of the software coordinate system with the palm facing up/down, as depicted in Fig. 4(a). The clinician subsequently selects the model and assigns it to the mesh-input component in Grasshopper 3D. Further, based on the fracture status, the clinician can draw two lines to define the immobility area from the top viewport. Line A is located on the side against the palm while Line B is located on the side close to the body. As previously mentioned, holes and deformations that occur between fingers could easily result in failure during subsequent construction of the basic covering surface. Construction of curves must, therefore, avoid generation holes and deformations near limb-model edges. Furthermore, thumb fixing is necessary in immobilization treatments; to address this concern, a procedure previously developed and published by the authors [19] was incorporated in this study, thereby facilitating the palm opening to fix the thumb [6, 22]. To serve as input to the finger-fixing situation, line A can cross the thumb web-space, as depicted in Fig. 4(b). Once the two curves are drawn, each could be assigned to the two input components in Grasshopper 3D and output to the next modeling stage.

#### Basic-surface construction

**Fig. 5:**
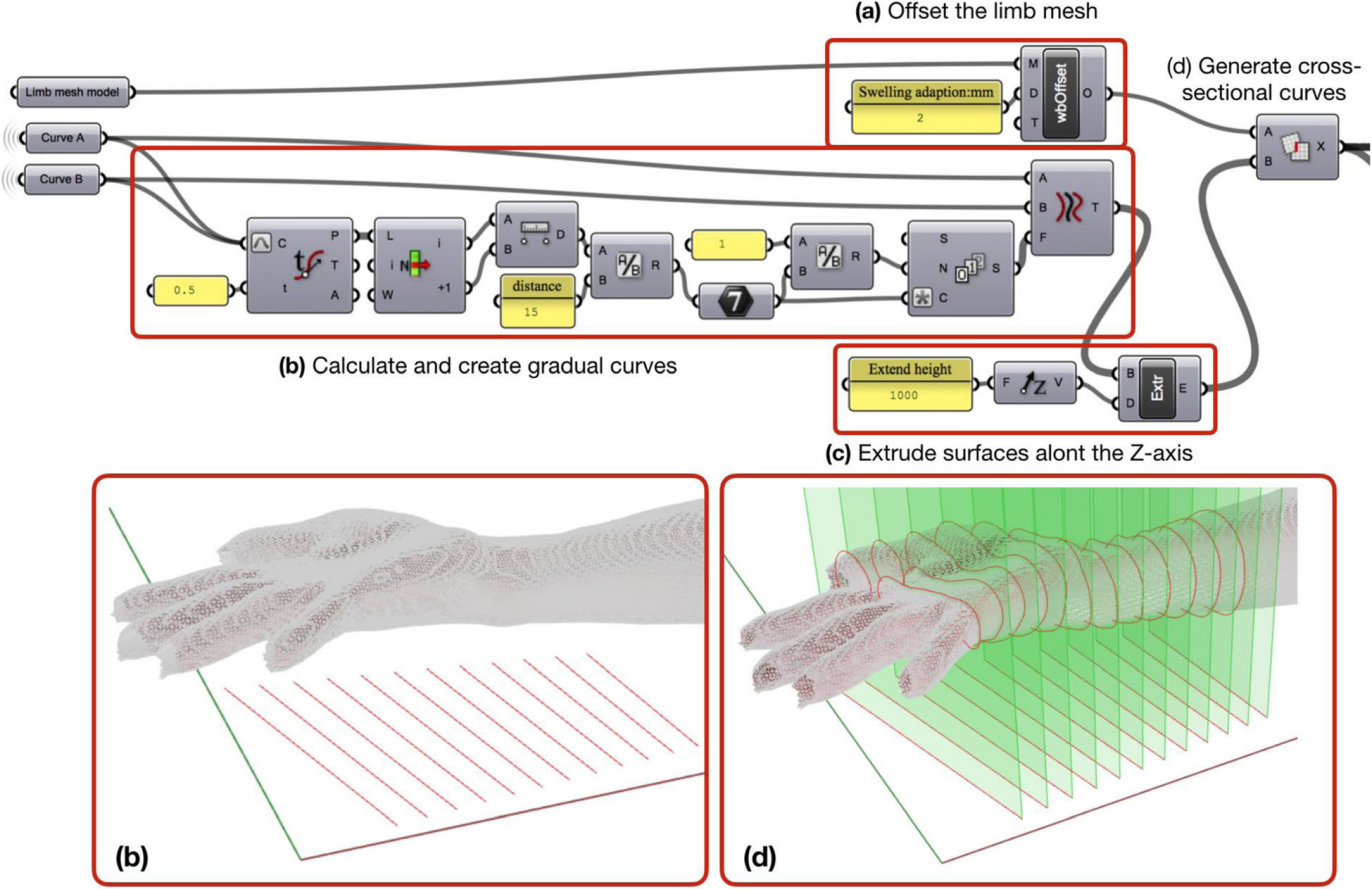
Generate cross-sectional curves. (**a**) Offset the limb mesh by 2 mm distance, (**b**) Calculate and create gradual curves, (**c**) Extrude surfaces along the Z-axis, (**d**) Generate cross-sectional curves

**Fig. 6:**
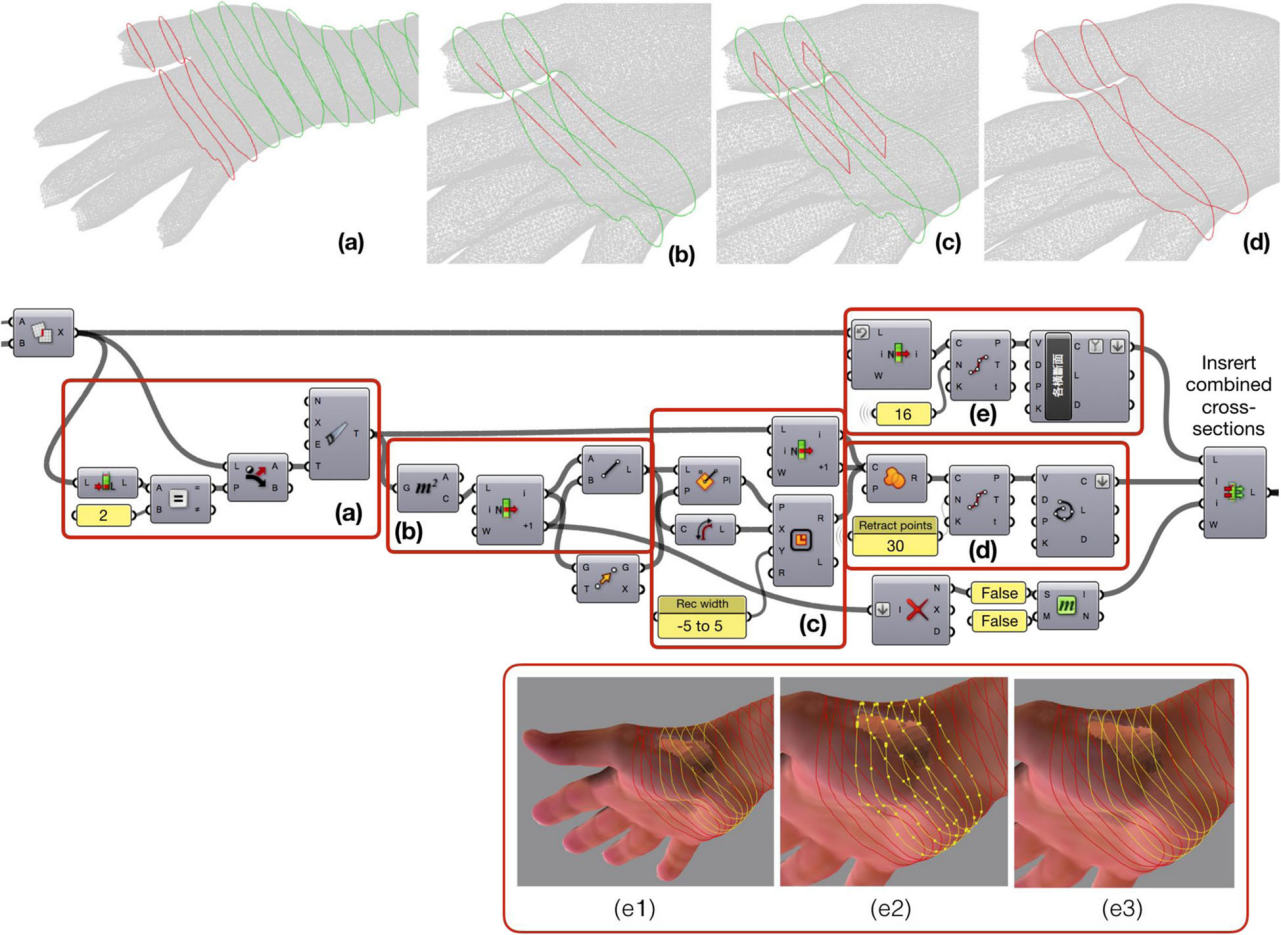
Regenerate and fix curves. **a** Detect dual cross-sections, if the amount geometry equals two. The first two sets, red curves are picked. **b** Connect the central points of cross-sections. **c** Generate rectangles connect cross-sections. **d** Combine rectangles and cross-sections. **e** Fix the scan flaws by extracting points and regenerating curves

**Fig. 7:**
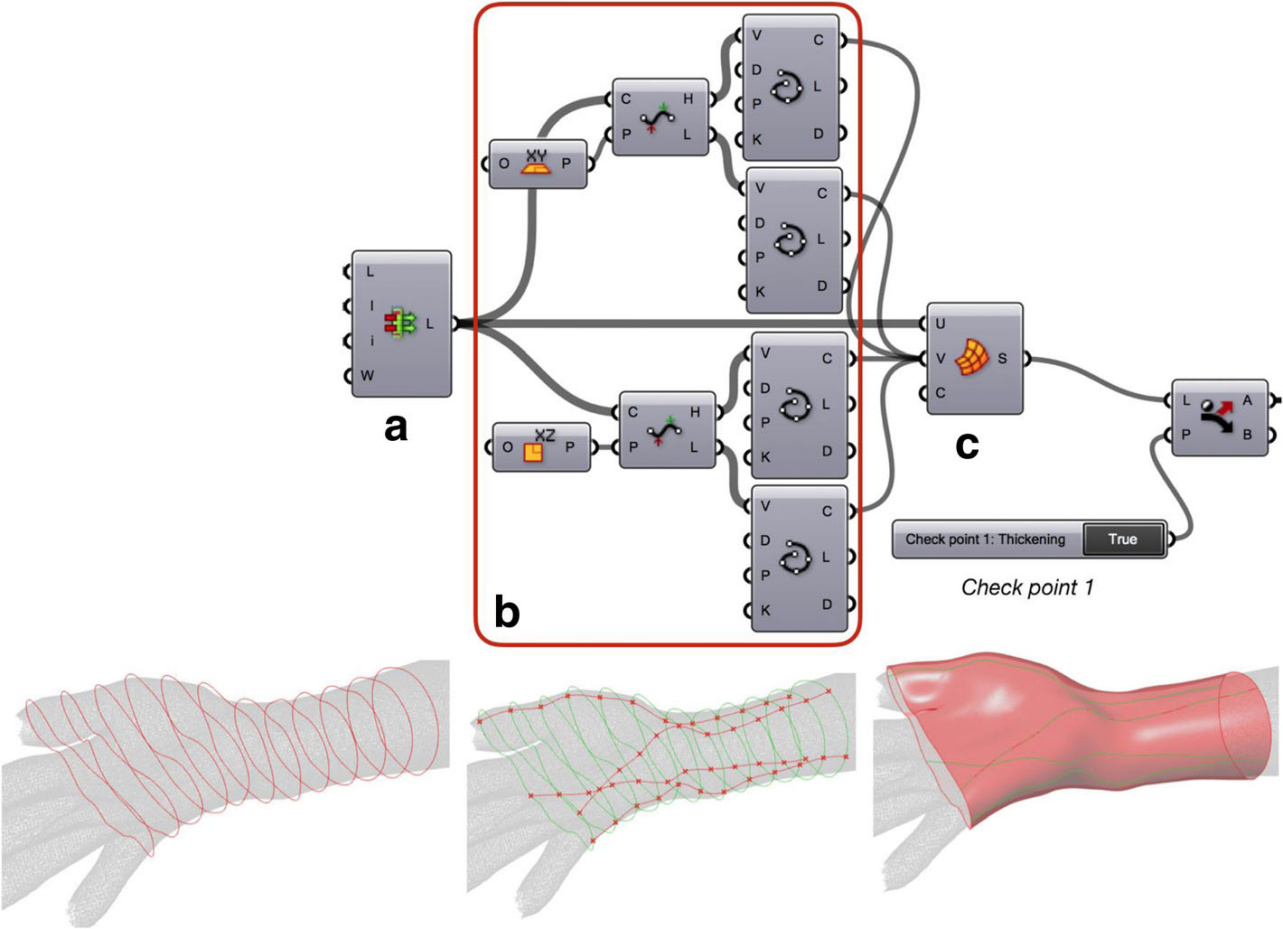
Generate covering surface. **a** Cross-sectional curves for U input of Network component. **b** Extreme points on XZ and XY planes of each cross-sectional curves are extracted to form curves for V input. **c** Parametric surface generated by Network component

Once clinicians complete the input stage, the system generates the basic surface as per the process depicted in Figs. 5, 6, 7. The analytical surface, which covers the affected limb, forms the critical foundation for the splint model. The clinician can examine the status of lines A and B projected onto the limb and the way they wrap around the limb from a perspective viewpoint.

For simulation of limb swelling, the mesh model is offset by approximately 2–3 mm (Fig. 5(a)) [14]. Several gradual lines are generated in the immobility area between the two input lines, as depicted in Fig. 5(b), and their distribution density is determined based on the splint length. The spacing between the lines, as observed in this study, was of the order of 1–2 cm. The density level was sufficient for displaying most arm features; in the case at hand, 12 curves were inserted. The gradual lines extend upwards, as surfaces along the Z-axis, to intersect with the limb and subsequently generate cross-sectional curves for the U input of the network component in Grasshopper 3D (Fig. 5(c, d)).

If line A is drawn across the thumb web-space, this implies that dual cross-sections of the palm and thumb may appear in few projections near line A. Network modeling, however, allows projections of only single cross-sections. A procedure was, therefore, designed to merge together dual cross-sections and fix the thumb by means of a small gap, as depicted in Fig. 6. Upon detection of dual cross-sections, a line would pass through central points located on separate cross-sections and offset on both sides with distance of 5 mm as a rectangle (Fig. 6(a, b, c)). A new shape would, therefore, be obtained, via combination of the rectangle and connected cross-sections, and subsequently smoothed by means of the “Interpolate Curve” command (Fig. 6(d)). The shapes, thus obtained, would replace dual cross-sections appearing in the U input of the network component, and the design of a slim gap between cross-sections can be used to fix the thumb (Fig. 6(d)).

However, there may exist modeling flaws in the arm scan, such as presence of a hole in Sample A, as depicted in Fig. 1(b), which could result in the presence of unclosed curves at the intersection; these curves could, in turn, generate serious distortions on the basic surface(Fig. 6(e1)). To fix this problem, 16 points extracted from the unclosed curve could be used to regenerate the closed curve through use of the interpolation command (Fig. 6(e2, e3)), and the corresponding repaired cross-sections could generate the entire covering surface. Such techniques [15, 16, 23] help overcome issues encountered during scanning. However, if the observed hole is large or if the immobilization area overlaps with the edge, there still exists a chance that the covering surface would be distorted. In such situations, the clinician can fix the problem by slightly moving the curves or simplifying them by using fewer defining points to avoid generation of a distorted surface.

After the cross-sectional curves are ready, extreme points corresponding to each section on the XZ and XY planes were extracted to form curves for the V input (Fig. 7(a, b)). The network component can generate a parametric surface within the immobilization region (Fig. 7(c)). Cross-section curves run along the U direction of the surface while long edges run along the V direction.

#### Division and thickness generation

**Fig. 8:**
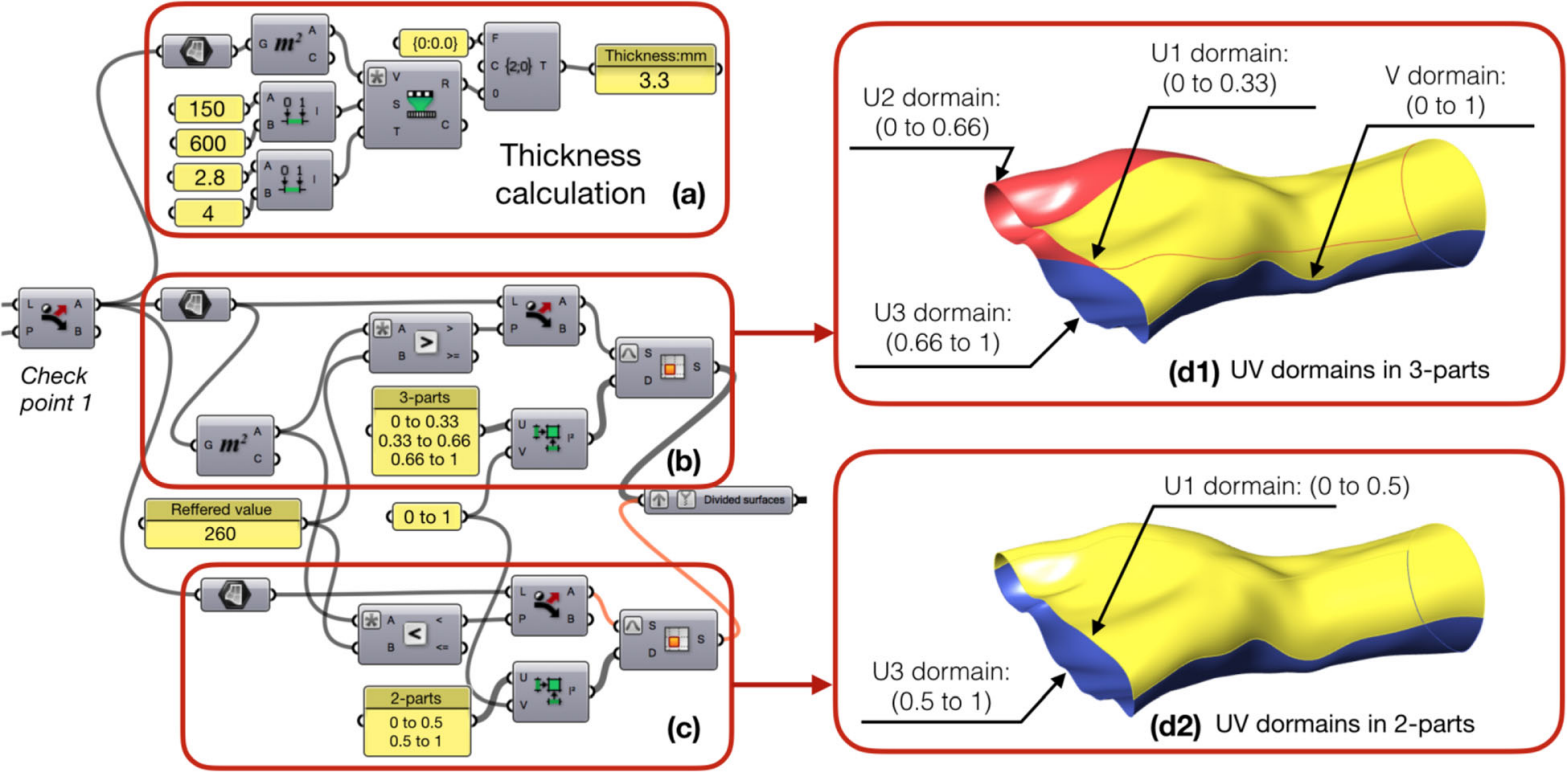
Thickness and surface division

**Fig. 9:**
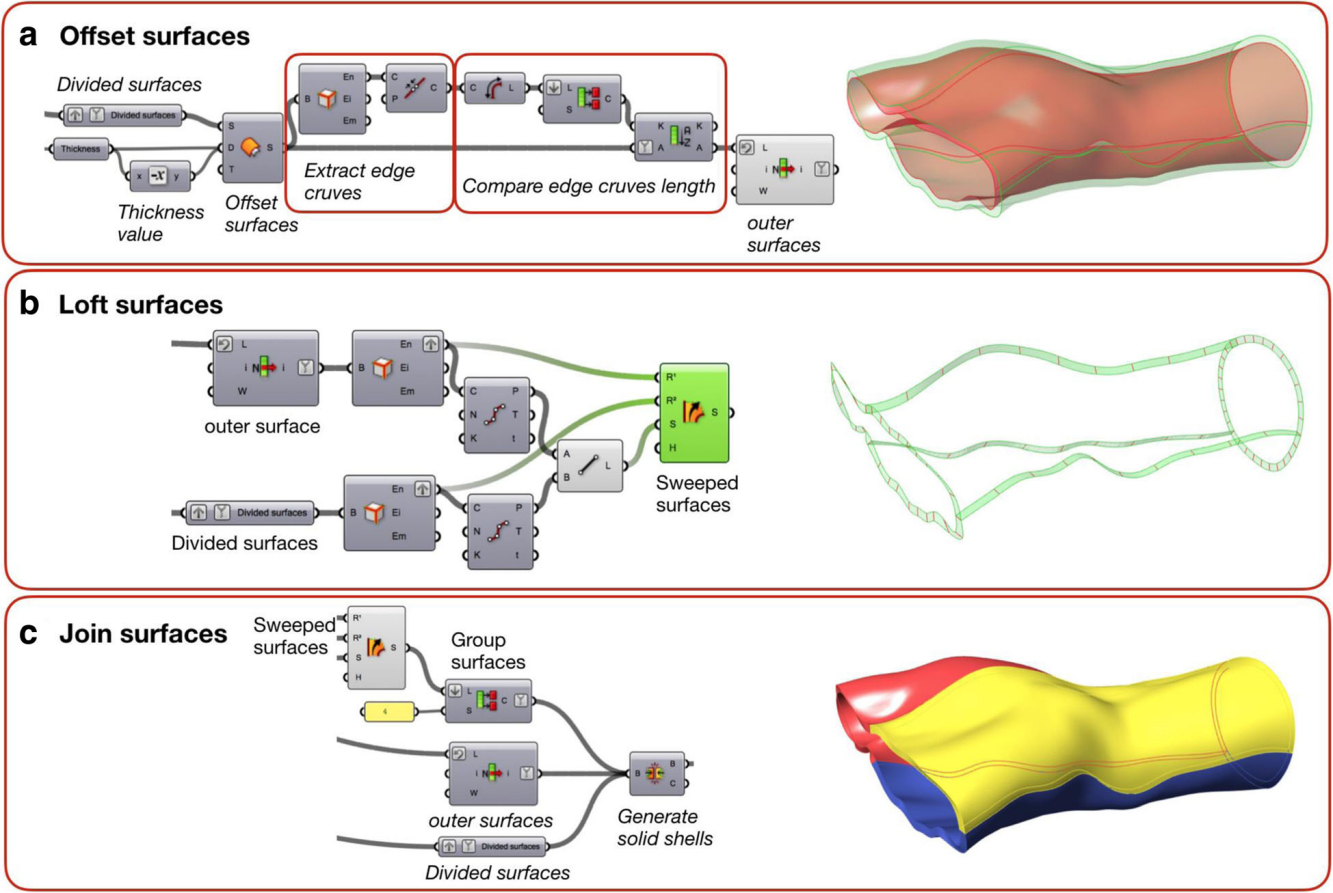
Generate solid shells. **a** Offset surfaces. **b** Loft surfaces. **c** Join surfaces

This step divides the covering surface into two or three surfaces covering the same area based on the overall model size. A shell with a certain thickness is grown over each surface (Figs. 8, 9). Thickness of the shell depends on the divided surface area and printing experience in order to attain the minimum required strength (Fig. 8(a)). Also, dividing the splint into two or three parts helps reduce the total printing time if multiple 3D printers are available. The dividing strategy is based on the trade-offs involved between the desired model strength and printing time.

- The wrist-splint default design is a two-part set, wherein the system evaluates the square measure of the basic surface and divides it into three equal parts if the total area is greater than a specified reference value, which in this case, was set as 260 cm^2^, as depicted in Fig. 8(b, c). The said reference value nearly equals the square measure of the covering surface of an adult palm. A larger splint used for ulnar-radius fractures is also divided into three equal parts. The system divides the edge length along the U-direction into three domains, as depicted in Fig. 8(d1, d2), and extracts isoparametric subsurfaces. Different colors are used to distinguish between the three surfaces depicted in the figure. If the splint area assumes a value nearly equal to 260 cm^2^, the clinician can adjust the size of the referred area to determine the portion.
- Splint thickness are calculated through use of the Remap component (Fig. 8(a)) per the splint area, which may be as small as a child’s palm or as large as an adult’s forearm, i.e., ranging from 150 to 600 cm^2^. Based on the area domain under consideration, the splint thickness ranges from 2.8–4 mm. This conversion, however, represents only a rough estimation, and accurate calculation of the surface thickness that provides sufficient strength to the model requires further mechanical validation.
- After determination of the surface thickness, peripheral surfaces are offset by the system, with respect to divided surfaces, by this distance. The edge-line of the two surfaces generates a band-shaped surface through use the “Sweep 2 rails” command. A solid shell is formed when the two surfaces are connected by means of the sweeped surfaces (Fig. 9(a, b, c)).

#### Lattice pattern and structure

**Fig. 10:**
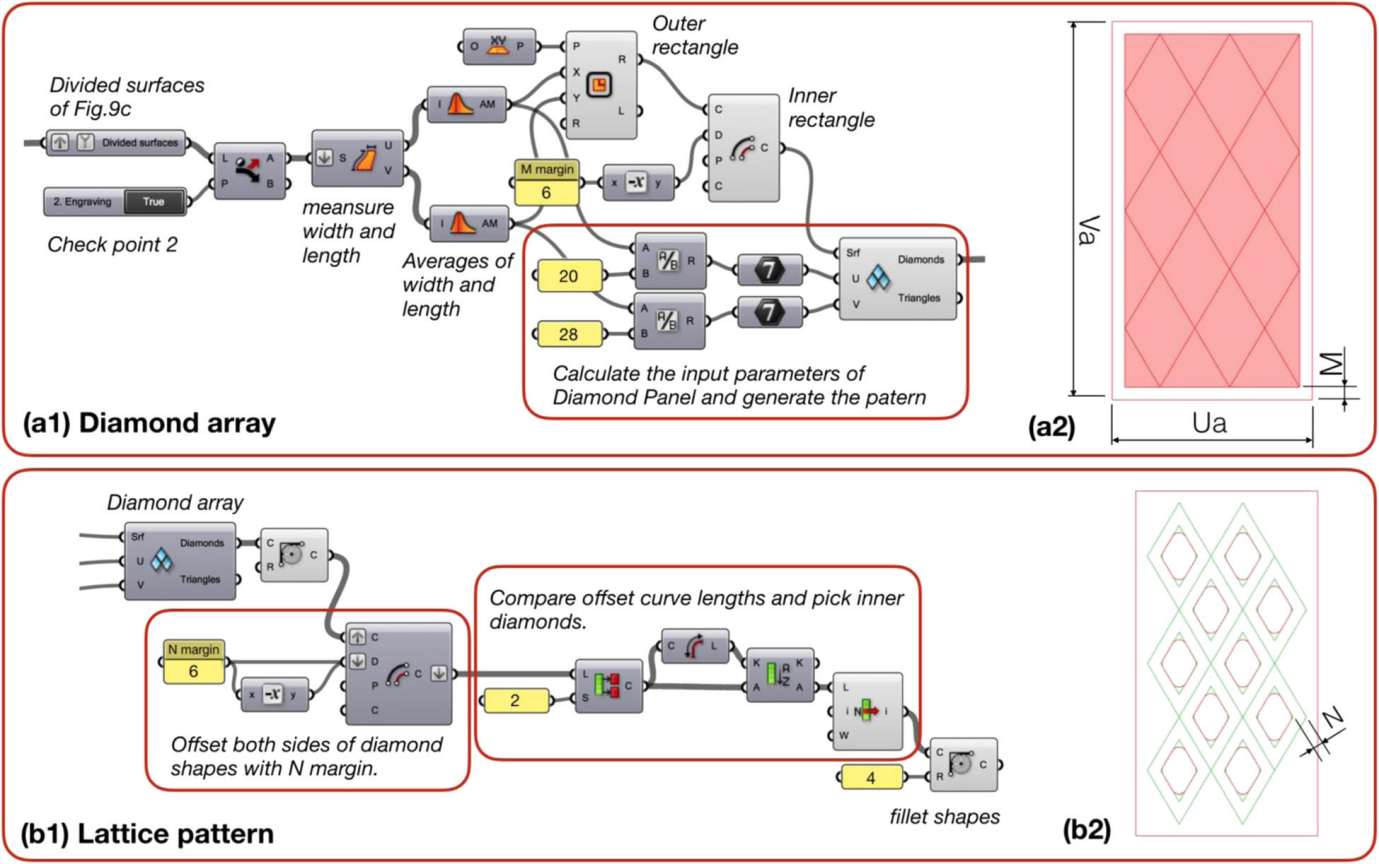
2D Lattice pattern—(**a**1) Program of generating diamond array pattern (**a**2) Diamond array pattern preview; and (**b**1) Program of generating structural pattern; (**b**2) 2D lattice pattern preview

In this step, a 2D preview of the lattice pattern (Fig. 10) is generated by the system and subsequently projected onto the shell.

**Fig. 11:**
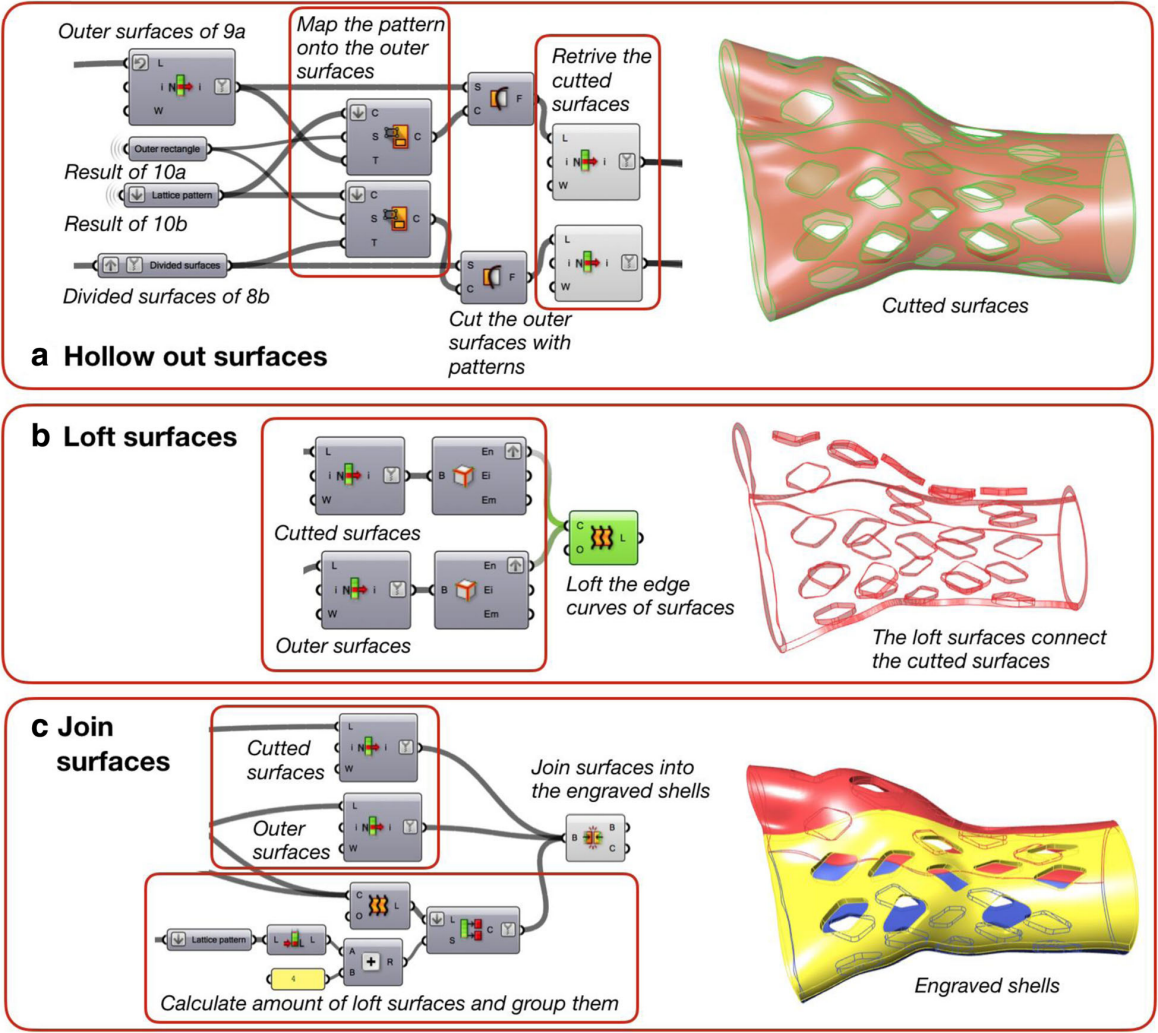
Engraving process **a** Hollow out surfaces. **b** Loft surfaces and connect cute surfaces. **c** Join surfaces. Mapping pattern on the shell; and (**c**) Engraved shells

- The system uses a diamond tessellation array to generate the lattice pattern, which—if the model is placed in the vertical orientation—serves to reduce both, the amount of support material consumed and printing time.
- In the 2D pattern preview, average lengths of the U and V edges—U_a_ and V_a_, respectively—corresponding to the three divided surfaces are used as the width and length, respectively, of a 2D rectangle (Fig. 10(a1, a2)), and offset an inner one with the M margin. After projection, the spacing from the M margin generates an external frame around the lattice structure, and the diamond tessellation pattern is generated within the inner rectangle.
- The tessellation pattern is generated by the “Diamond Panel” component in Grasshopper 3D and required inputs—the U and V divisions. The diamond array within the pattern is determined by how the rectangle is divided by oblique lines along U and V directions, and amounts of diamonds are in proportion to Ua and Va of rectangle. In the sample depicted in Fig. 10(a1), U and V divisions are given by U_a_/20 and V_a_/28, respectively. These coefficients are, however, not absolute, and the rule is to reduce the support material generated within the lattice structure during printing. U/V division numbers used in this example were set as five and seven, respectively. Each diamond in the tessellation was offset by the N margin and provided width to the structure truss.
- Figure 11(a) depicts the 2D pattern projected onto the inner and outer surfaces of each splint shell. The pattern could be used to cut holes in the lattice. The loft command can be used to create green surfaces between two-hole edges, as depicted in Fig. 11(b). Finally, all remaining surfaces, including lofted ones, could be joined together to form a solid latticed shell (Fig. 11(c)). If the system fails to engrave the shell, values of parameters U and V in the divided area could be decreased to enlarge pattern holes, thereby avoiding generation of tiny holes that cause operation failure.

#### Rounded-edge and screw-seat generation

**Fig. 12:**
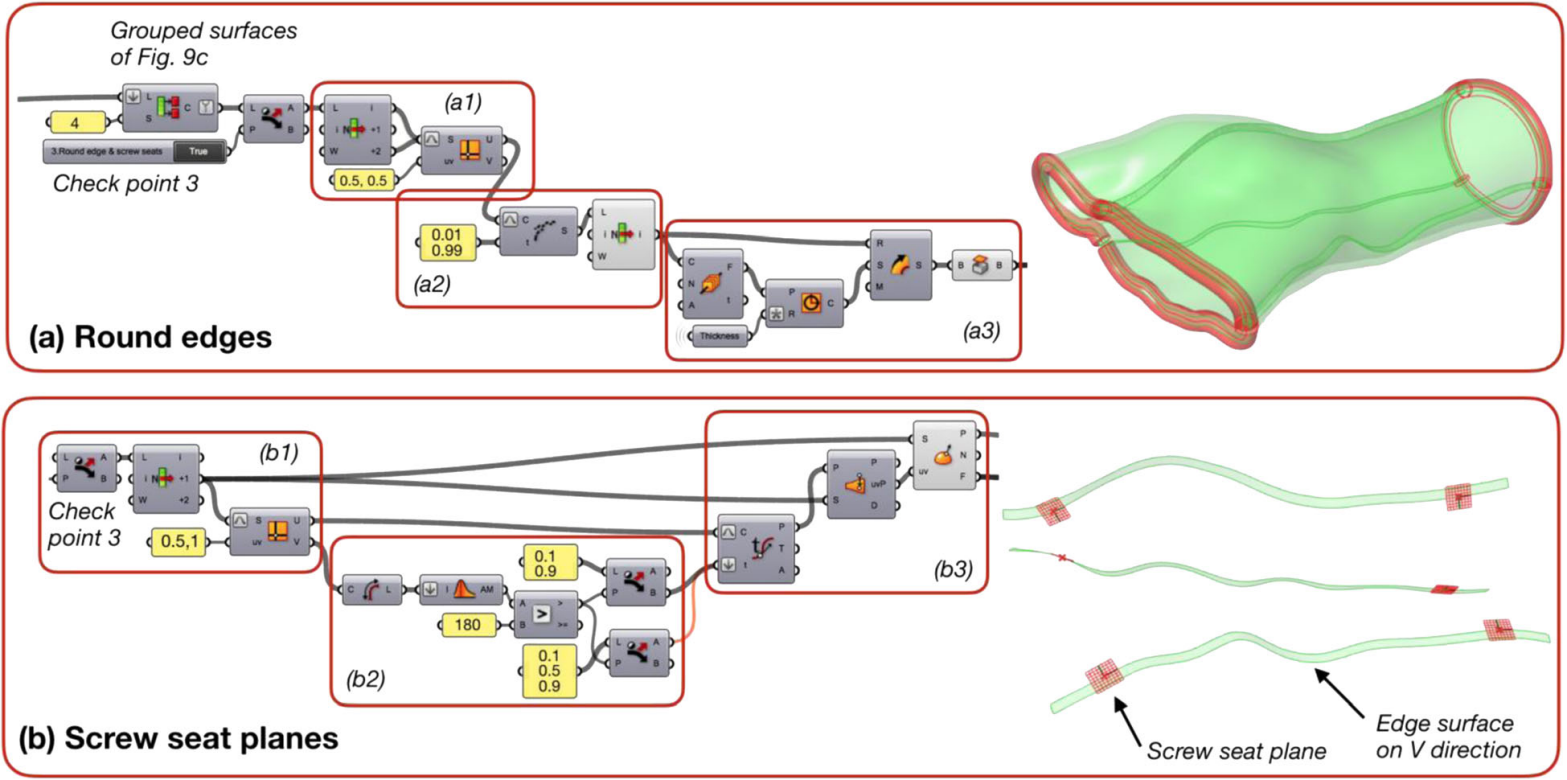
Generation of round edges and screw seats—(**a**) Round edges. (a1) Extract isocurves from front and backward edge surfaces, (a2) Extract main segments of isocurves on U direction, (a3) Generating round edges on the splint edges by Sweep command; (**b**) Screw seat planes. (b1) Extract isocurves of edge surfaces on V direction (b2) Meansure V edge lengths for Judgement of adding additional screw seats; (b3) Generate screw seats onto edge surfaces

This is the last step involved in splint-model generation, and generates two important features—rounded edges, for preventing skin abrasion, and M3 screw seats, to facilitate the assembly of different splint parts (Fig. 12). The program depicted in the figure corresponds to that of the sample comprising three parts.

**Fig. 13:**
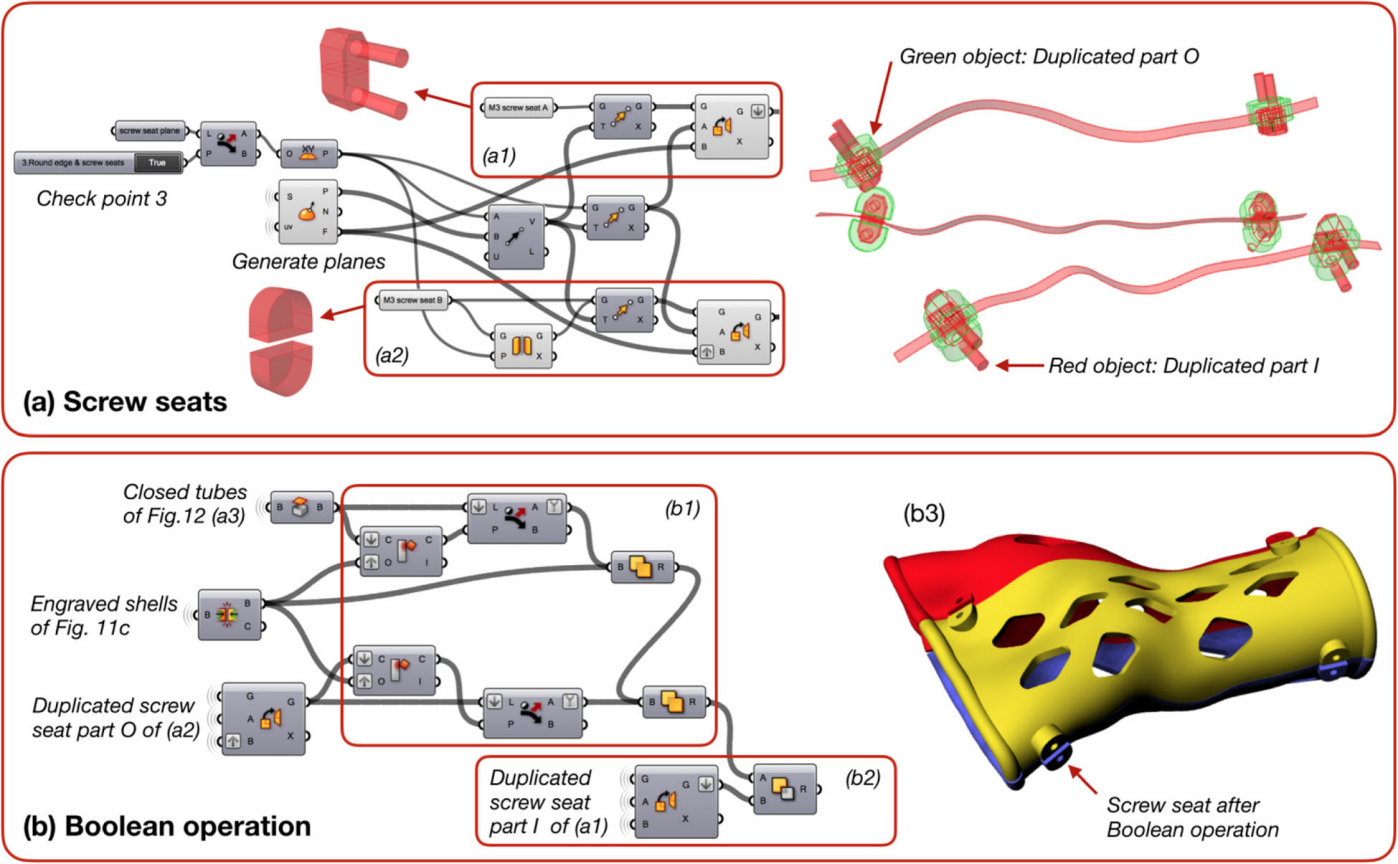
Generation of screw seats and boolean operation. (**a**) Screw seats (a1) Screw seat part I is embedded in the Brep components and duplicated to the planes (a2) Brep component of Part O is duplicated on the same planes; (**b**) Boolean operation (b1) Union Boolean of part O, engraved shells and round edges (b2) Boolean subtraction of duplications of part I and engraved shells (b3) Final splint model

- For the round edge, two tubes were developed along the U direction edge of the splint surface through use of the “Sweep” command (Fig. 12(a)). Two isocurves of edge surfaces on the U direction of each splint were extracted to serve as the sweep path, and the main segment of isocurves are extracted with the curve domain 0.01–0.99 (Fig. 12(a1 and a2)). Perpendicular planes at the ends of the edge lines were simultaneously defined. Two circles were drawn on these planes, with diameters roughly large than the splint thickness about 1.5 mm, to serve as tube cross-sections (Fig. 12(a3)).
- The splint was assembled by fastening several M3 screws. Several planes are set for placing the screw seats to precise positions on edge surfaces of V direction, The screw seat plane was duplicated from the original position onto two points on isocurves with parametric position 0.1 and 0.9 on each V edge surface, as depicted in Fig. 12(b1, b2). If the V-edge length exceeded 180 mm, an extra screw seat plane was added at midpoints along V edges.
- An embedded model of the M3 screw seat, part O and I were installed on the XY plane (Fig. 13(a1 and a2)) and duplicated onto the planes. The screw seat was created via Boolean subtraction of two parts, wherein the part I was subtracted from the part O, thereby creating space for containing screw nuts. The screw sets work by constraining nuts, since the screw threads were too tiny to be printed in the prototype. Finally, each splint shell was combined with two tubes and 4–6 screw seats through use of the Boolean union and difference commands (Fig. 13(b)).

The entire splint-modeling process of the system can be described by means of the above steps. The proposed system can, therefore, assist the clinician in generating a feasible splint model within few minutes. However, system failures are still possible. As such, it is important to explain to the clinician the operating principles of the system and remedial measures to be employed during different stages in the event of a system failure.

#### Mechanical strength testing

**Fig. 14:**
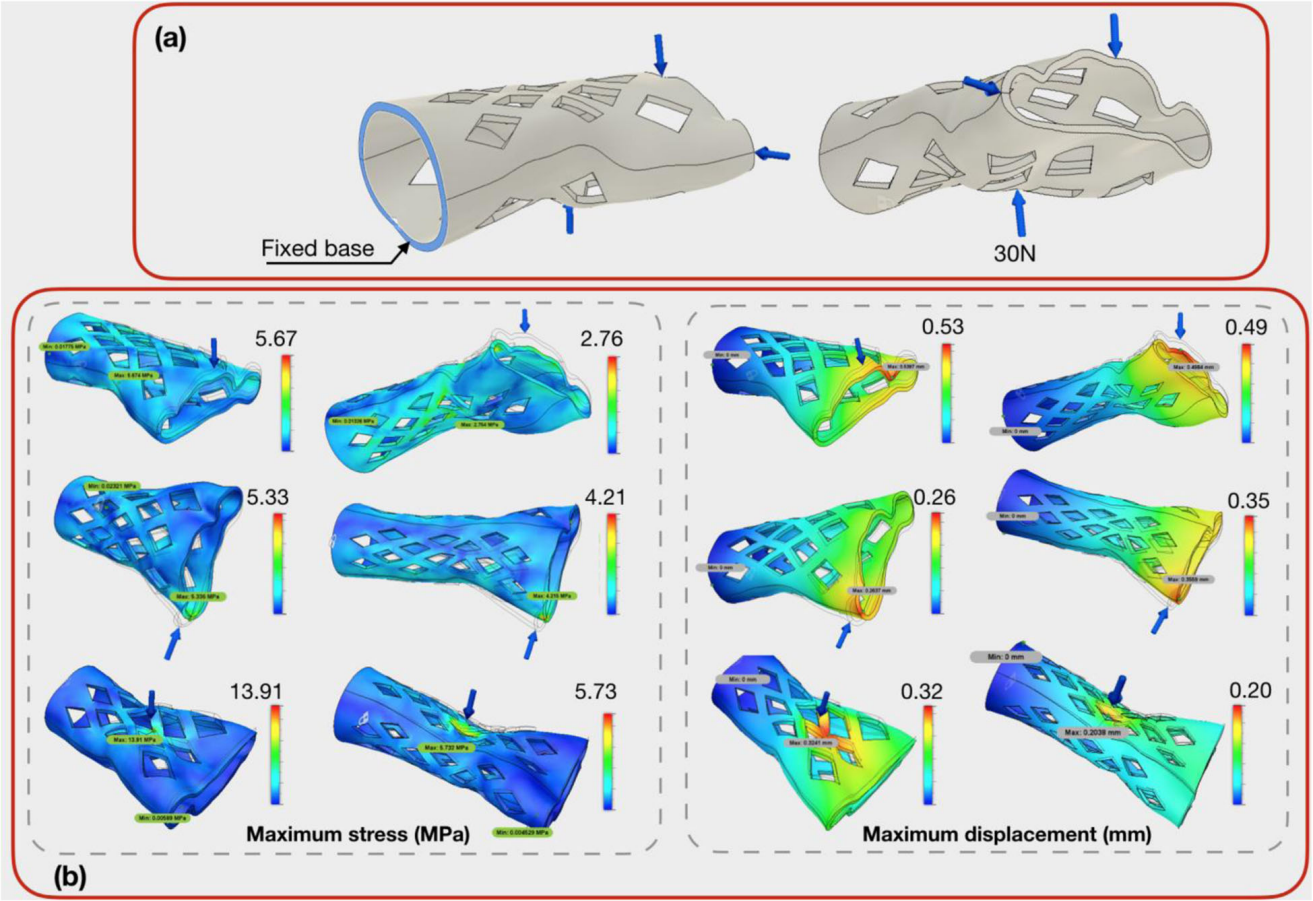
FEA test and result. (**a**) Structural constraints and load of simulation setting; (**b**) FEA result, von Mises stress value, and displacement under applied splint loading. The maximal Von Mises stress value is 13.91 MPa and maximal displacement is 0.53 mm, both values happen on the 2-parts splint

A finite element analysis (FEA)-based static stress simulation was performed to test the engineering strength of generated splint designs against predictable forces. Two- and three-part splint designs based on the previously mentioned limb sample were imported into Fusion 360 (Autodesk) to perform an independent simulation. Acrylonitrile butadiene styrene (ABS) was used as the material to define material properties in the simulation setup with Young’s Modulus (E) and Poisson Ratio (v) values of the order of 2240 MPa and 0.38, respectively [22, 24]. Splint parts were assembled using screws to resemble a patient’s limb in a state of immobilization to facilitate their import into the simulation as a single object (Fig. 14(a)). The splint model was simplified to facilitate simulation calculations by removing round edges and screw seats. The structural constraint was set with the proximal edge marked as the fixed base. The thickness and lattice structure density of splint were varied in accordance with the splint size in the modeling program. The 2-part splint was generated with a thickness measuring 3.2 mm and a 6 × 9 diamond array on the structure of each part. Likewise, the 3-part splint measured 4-mm thick with a 9 × 5 diamond array on each part structure. A structural load of 30 N was applied on the distal edge of the splint and lattice-structure area along three directions separately to simulate possible hits and stresses that may unintentionally occur during the recovery period. Results obtained this simulation along with maximum von Mises stress values and displacements are depicted in Fig. 14(b) demonstrating sufficient strength of the proposed splint designs.

#### Splint fit investigation

**Fig. 15:**
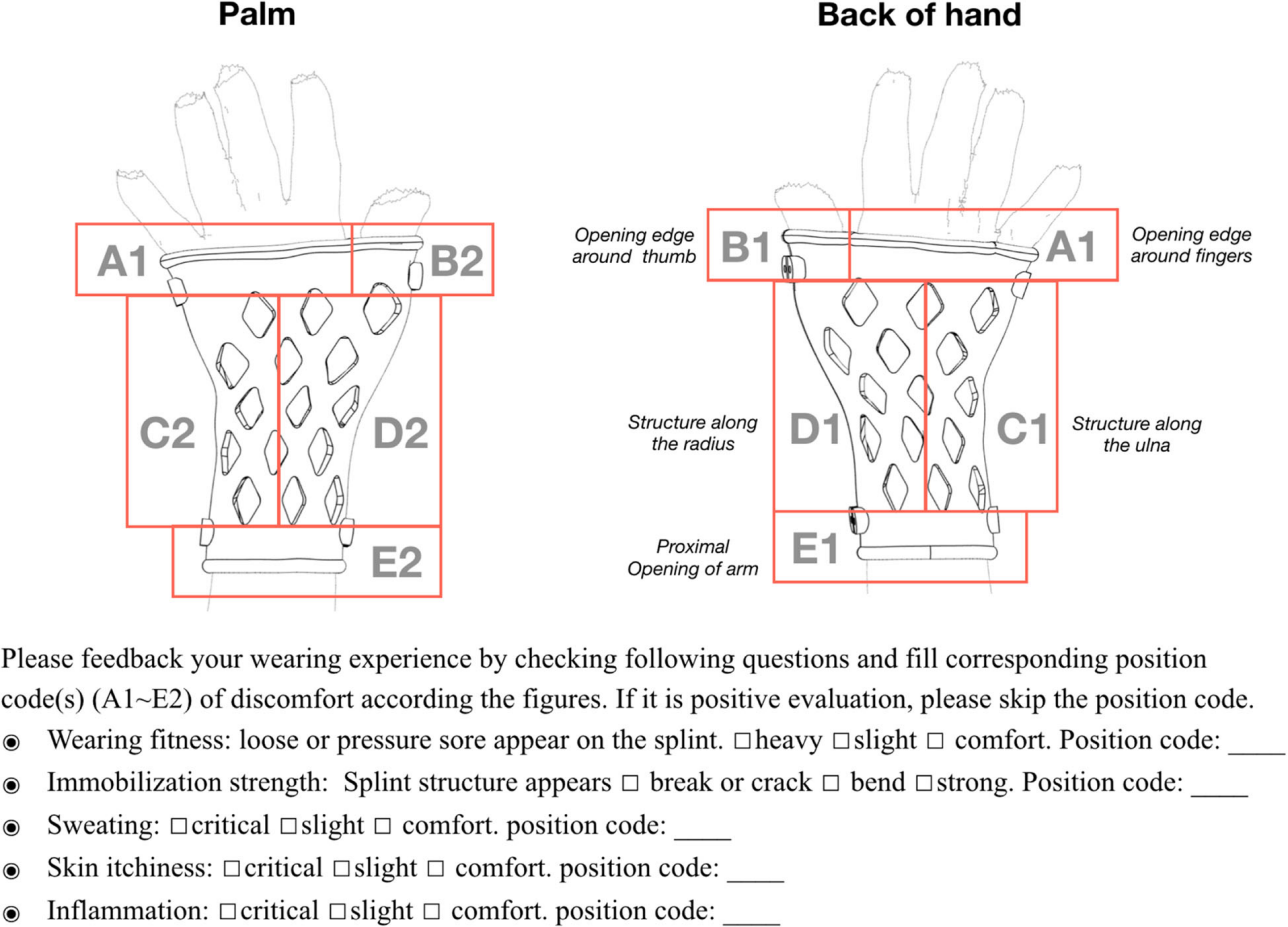
Splint fit questionnaire

A 3-h wearing experiment was performed on health volunteers within an enclosed environment, and a 3-level-scaled questionnaire was designed to assess their experience of wearing splints designed using the proposed system. The purpose of this experiment was to determine possible faults in the splint design, thereby facilitating preparations for performing further studies involving more wearers, longer durations, and larger immobilization regions. Five performance indicators were considered in this investigation, including wearing fitness, immobilization strength, sweating, skin itchiness, and inflammation, and a set of wrist figures were attached in the questionnaire for the wearer to mark specific positions of the above-mentioned discomforts, including locations of pressure sore, splint-structure cracking, sweating, and inflammation (Fig. 15).

## Results

### Time required for splint-model generation

**Fig. 16:**
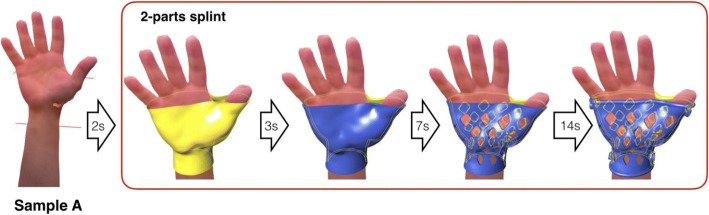
Required computation time(s) for each stage during design of Sample A

Five sample splint designs for actual fractures were generated in this study. The samples comprised a wrist splint assembled in two parts and a larger splint—that covered the palm and forearm—designed in three parts, as depicted in Fig. 2. Curve drawing and providing splint geometries as input to Grasshopper 3D usually takes 1–2 min depending on the clinician’s diagnosis. The time required for model calculation at each stage for Samples A and E is included in Fig. 16, wherein it has been marked between model results. In the 2-part splint process, generation of the covering surface consumed 2 s while that of solid shells of required thicknesses was completed in 3 s, and so on.

The total time spent on the five samples was recorded and has been listed in Table. 1. Most stages took little time (few seconds), whereas the last stage, wherein round edges and screw seats were generated, lasted approximately 8–25 s. As expected, the design of the 3-part splint was observed to be slightly more time-consuming compared to the 2-part splint. The overall design process, including curve drawing and geometry input lasted roughly 2–3 min, in most cases.

**Table 1:** Time required for splint-model generation during each stage of five samples

		Required time (seconds) of calculation in each stage				
Sample	Parts amount	Covering surface	Offset solid shell	Lattice structure	Round edge & screw seats	Total
A	2 parts	2	3	7	14	26
	3 parts	2	4	10	25	41
B	2 parts	3	3	4	9	19
	3 parts	2	3	7	18	30
C	2 parts	2	3	3	8	16
	3 parts	2	2	4	13	21
D	2 parts	2	3	3	11	19
	3 parts	2	3	6	18	29
E	2 parts	2	3	7	18	30
	3 parts	2	4	8	20	34

### Lost-mesh fixing performance

**Fig. 17:**
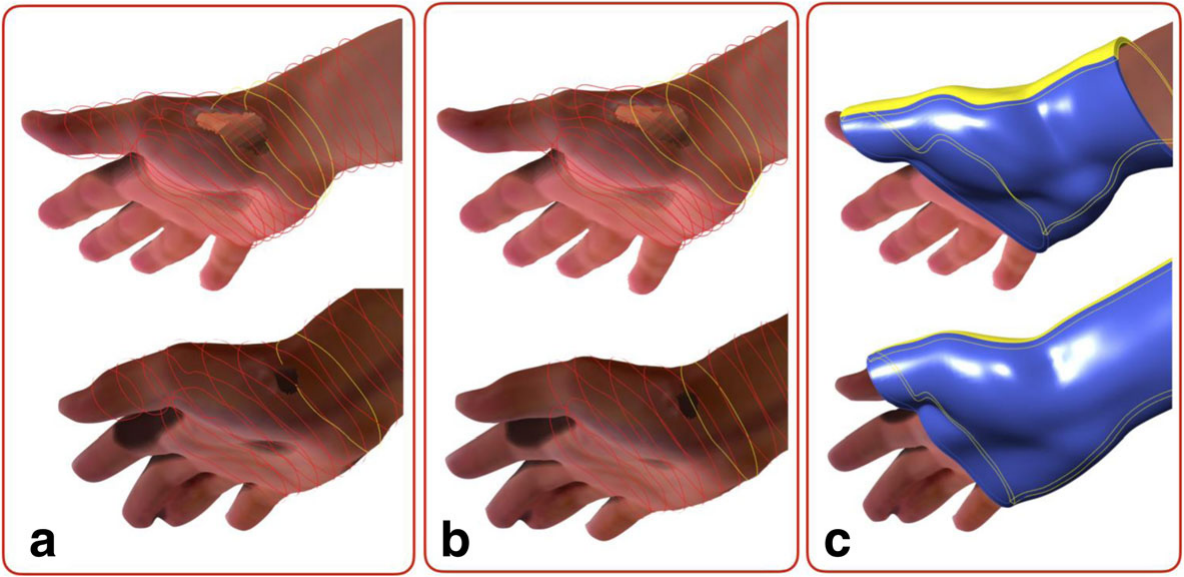
Result obtained upon fixing lost meshes in samples A and E—(**a**) Holes in samples A and E; (**b**) Regenerated closed cross-sections; and (**c**) Complete shells

During simulations, a small area of the mesh in samples A and E was lost owing to deficient illumination during the scanning process. The samples, therefore comprised holes measuring approximately 2 × 4 cm^2^ and 1.5 × 2 cm^2^, respectively, and located in the immobilization region, as depicted in Fig. 17(a). Presence of holes lead to breaking of a few cross-sections marked by yellow curves. In both samples, the system extracted points from unclosed curves, thereby automatically regenerating closed cross-sections (Fig. 17(b)). As depicted in Fig. 17(c), the covering surface generated the complete shell; during subsequent fabrication and user fitting, the repaired area did not impact the splint’s fitness and/or comfort.

### Prototype fabrication

**Fig. 18:**
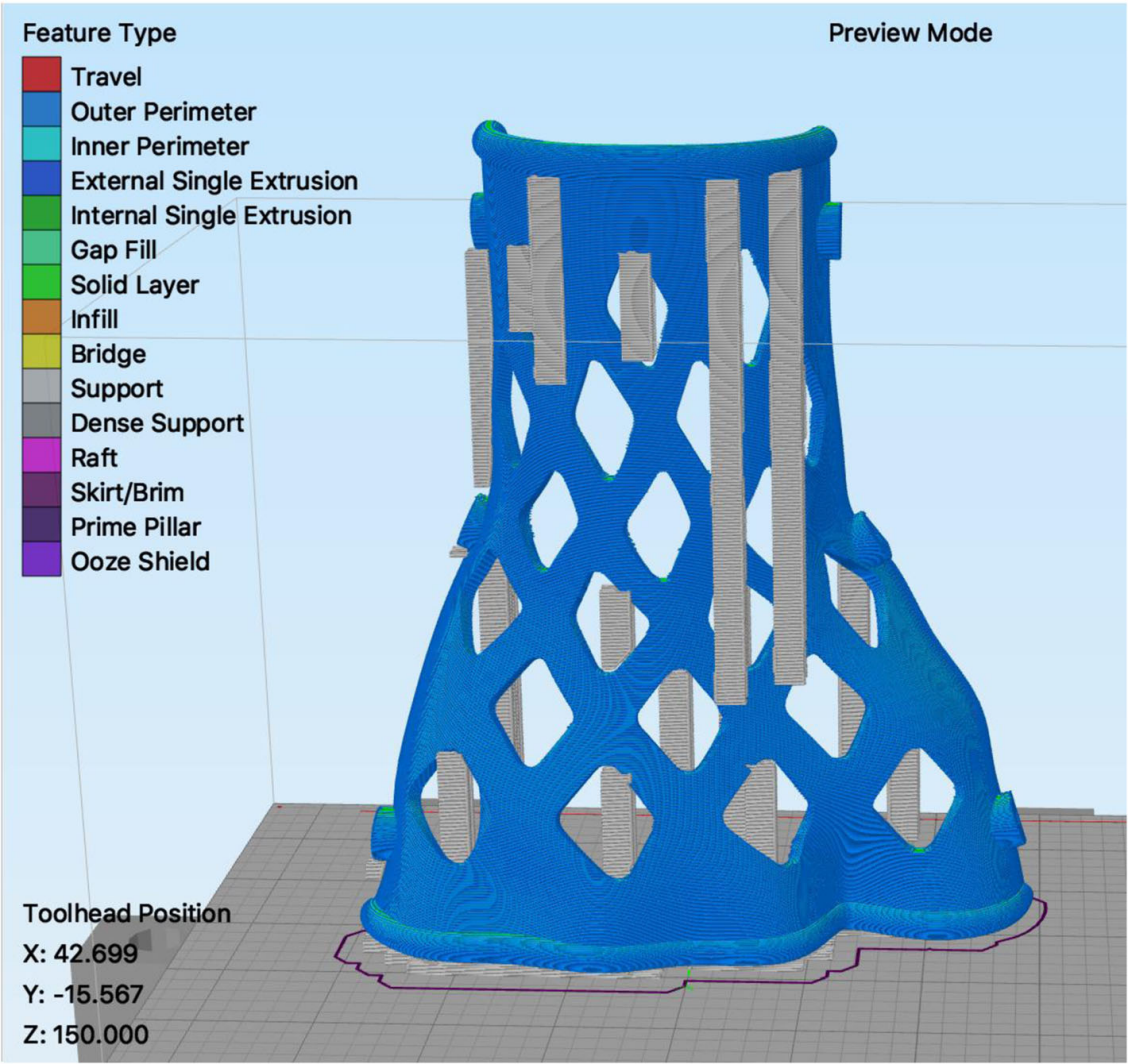
Printing placement in slicer software and printed prototype

Here, the time required to print the five samples was calculated. After exporting STL files, the Simplify 3D 4.0 (Simplify 3D) [25] slicer software was used to calculate the printing time under identical settings. All splints were printed at a standing posture, as depicted in Fig. 18, thereby conserving support material and saving printing time.

**Fig. 19:**
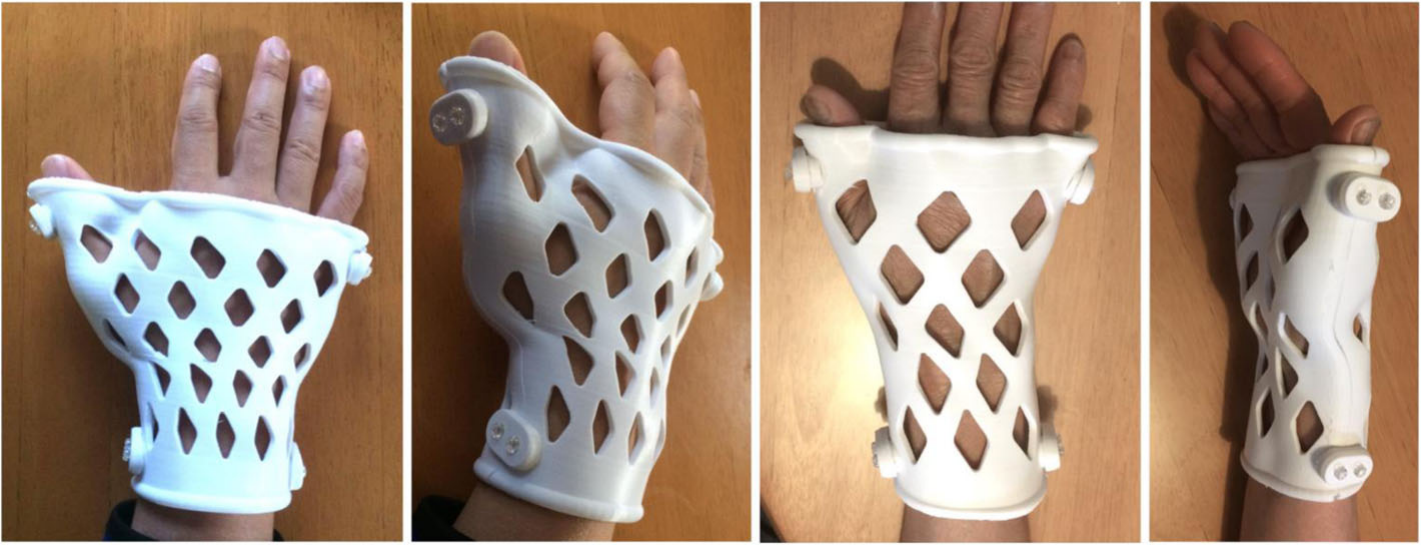
Printed splint prototypes for splint-fit experiment

Although the splint was divided into several parts occupying equal volumes, differences in the shape and consumed support material of these parts were observed to cause minor inconsistencies in the total printing time. The build time, splint weight, and height statistics of all splint parts are listed in Table 2. The longest printing duration corresponding to a given splint part indicates the time at which the part could be considered ready for installation on a patient. The said time is usually of the order of 2 h to less than 5 h depending on the splint height, and the total splint weight ranges between 62 and 194 g. Qidi Tech 1—commercial fused deposition modeling 3D printer (QIDI Technology)—and ABS material (tensile strength: 29 MPa) were used to print the splints, and two printed splints are shown in Fig. 19. The generated splints did not need require further finishing after the supports were manually removed.

**Table 2:** Fabrication data statistics, including the printing time, weight, and height of all the splint components calculated by the slicer software

Sample	part no	2-parts splint			3-parts splint		
		build time (h:m)	weight ( g )	height ( mm )	build time ( h : m )	weight ( g )	height ( mm )
A	1	2:42	44	129	4:28	64	259
	2	2:59	53	129	4:18	57	256
	3				4:35	73	261
total weight			97			194	
B	1	2:20	33	130	3:26	46	205
	2	2:22	36	131	3:16	37	206
	3				3:29	47	209
total weight			69			130	
C	1	2:42	41	158	3:55	47	241
	2	2:49	43	159	3:44	45	235
	3				3:52	43	245
total weight			84			135	
D	1	2:10	30	128	4:27	54	275
	2	2:14	32	128	4:31	53	281
	3				4:34	56	277
total weight			62			163	
E	1	3:38	58	178	4:12	52	257
	2	3:36	62	177	4:13	51	255
	3				4:10	56	257
total weight			120			159

### Splint-fit investigation

Five 2-part wrist splints were tested by health volunteers involved in splint-fit investigation. The wearers did not demonstrate any actual fracture symptoms; therefore, no offset operation for swelling adaption was performed during the design of these splint models. Results of the questionnaire answered by wearers are listed in Table 3, and no critical discomfort was reported. The splint features were customized based on 3D scan data with a view to provide comfortable fit to wearers’ limbs. Furthermore, the lattice structure that covered majority of the limb area was observed to improve ventilation, thereby preventing the wearers from facing issues related to sweating. Minor pressure sores and skin itchiness around position C1, the styloid process of ulna were, however, reported in three cases. These were observed to be caused by friction between the skin and hole edges within the lattice structure. To prevent hole edges of lattice structures from contacting the skin near bony regions, the lattice pattern must be made adjustable during the modeling process.

**Table 3:** Statistics result of marked times on each splint area based on the marked positions on questionnaires. The discomfort feedback of “Critical” is marked as ●, and “Slight” is marked as ○. Feedback of “Comfort” remained as blank. No critical discomfort was reported

	Indicators				
Position Code	Fitness	Structural Strength	Sweating	Skin itchiness	Inflammation
A1					
B1				○	
C1	○			○○	
D1					
E1					
A2					
B2					
C2			○		
D2		○			
E2				○	

## Discussion

Based on the proposed approach and subsequent verifications preformed in this study, a detailed description of the performance has been provided as well as related limitations have been discussed and addressed.

### Scan-flaw rectification

With currently available scanning technologies and limited compliance of the patient in terms of maintaining the required posture during scanning, presence of holes and deformations within the limb model is a major problem. The proposed system has the potential to remedy these flaws through use of a simple programmable modeling technique sans use of high-accuracy scanners and complex post-production procedures. The clinician can, therefore, ignore the presence of small holes that occur during the scanning process, thereby finishing the task much faster. With shorter scanning durations, patient trembling can be avoided. The proposed digital design technique simplifies the scanning process for clinicians and eliminates the need for them to learn additional post-production tools. However, the effect of the hole-filling function depends on the scale of gaps formed on unclosed cross-sections. If the gaps are large or if certain detailed features are located at these gaps, the command to fix concerned cross-sections via interpolation cannot be used, and the original shape cannot be accurately recreated. Indeed, clinicians must be aware of such a possibility.

In addition, computed tomography (CT), which captures medical images in the digital imaging and communication (DICOM) format has, at times, been utilized in fracture treatments—to facilitate further investigations in cases concerning hard-tissue traumas—and can serve as an alternate source for obtaining accurate external 3D surfaces of injured limbs sans the defects involved in the original 3D-scanning process. Through use of medical-image-viewer software, DICOM files could be transformed into STL models and provided as input to the proposed system for splint design, with the limb model appearing as a single object in the STL file to satisfy input requirements. However, CT imaging involves higher costs compared to 3D optical scanning and prescription necessities depend on the clinician’s diagnosis.

### Rapid design and training

Most CAD software are designed for creation of endless geometric diversities; however, splint design is a regular task involving generation of a model that exhibits specific features. Based on extant studies [3, 14], massive manual-modeling steps involved in the splint design and initial scanning tasks generally require up to 20 min to 3 h of completion time, which through use of the proposed technique, can be reduced to approximately 2–3 min.

The proposed programmable modeling tool is a key factor enabling the system designer to reduce the aforementioned manual operations in CAD to a few steps. The said tool also automatically performs required computations, thereby directly providing parameters of interest to the clinician, so that the splint design process could be completed within few minutes without the need for complex operations and/or training. The division-control method enables the system to effectively perform computations and allows clinicians to monitor splint generation at each stage. Basic viewport navigation, curve drawing, and parameter modification are the only skills required on part of the clinician, and these can be taught and/or memorized within 20 min. However, if the scanned model itself is of a poor quality, the clinician is more likely to obtain invalid results. This, in turn, would require more time to test different parameters and input curves. Nevertheless, if the solution fails, the 3D-scanning process can be repeated.

### Fabrication-time reduction

Presently, the printing process occupies the most time involved in the overall design and production of 3D-printed splints—approxiamtely10 h, in general. The proposed study, demonstrates the possibility of reducing the printing time to a few hours through use of the modeling technique, wherein the system divides the splint into two or three compassable pieces and simultaneously generates all parts using multiple 3D printers. The volume of support material utilized during printing can be economized via optimum model placement on the printing bed. Additionally, the structural thickness can be adjusted in accordance with the splint, area and corresponding computation can be embedded within the system. Surface areas of the 10 splints listed in Table 1 were automatically generated by the system after due calculation of their thicknesses and lattice-structure densities. However, attainment of the required splint strength requires further mechanical validation for revising parametric calculations concerning the thickness and lattice pattern.

### Swelling acclimation and comfort

**Fig. 20:**
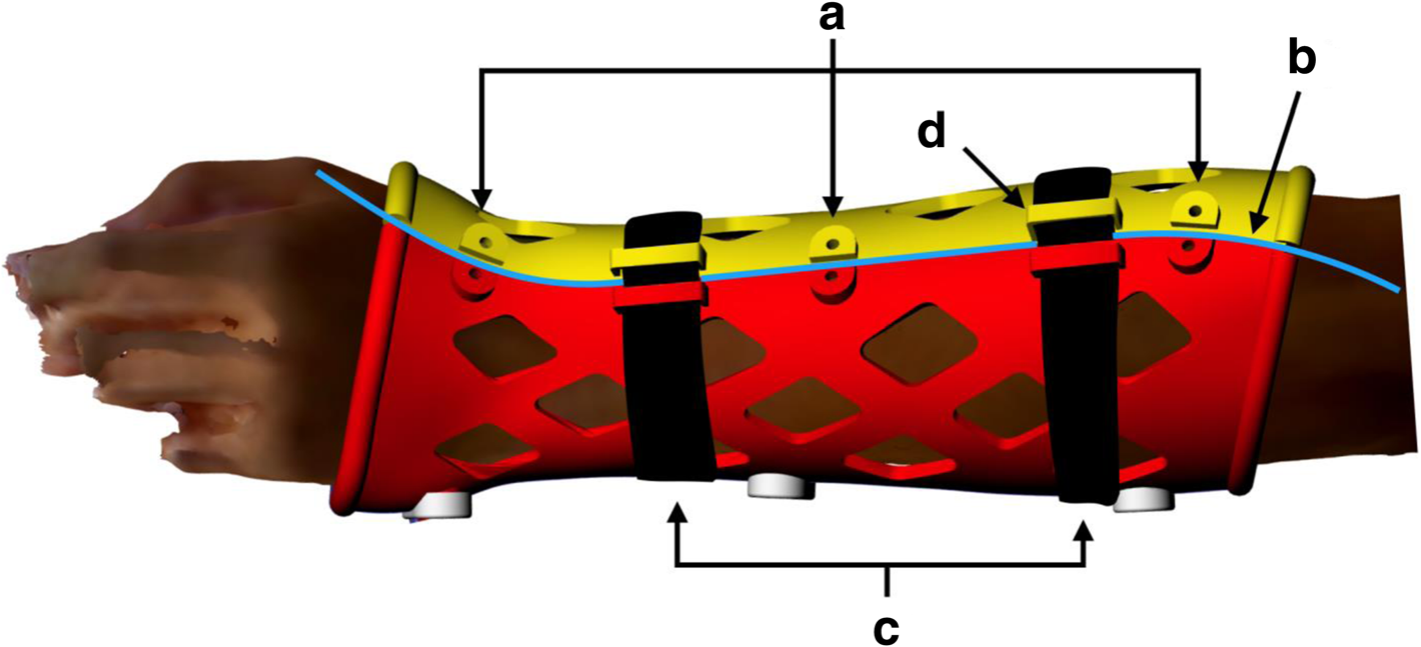
Solution to swelling accommodation—(**a**) Screw remove from gap; (**b**) Gap opening; (**c**) Velcro straps; and (**d**) Strap rings

Overcoming inflammations and swellings that occur over the course of fracture treatments is a common issue that must be accounted for during splint design, as prescribed by Fitch [1]. Splints designed using the proposed system are rigid and fastened by screws; hence, by removing a set of screws along one of the gaps, the splint can be rendered flexible to accommodate limb swelling, as depicted in Fig. 20(a and b). Two Velcro straps were employed for fastening the splint and adjusting its fit (Fig. 20 (c)) [3]; strap rings could be generated by the system—in a manner similar to placement-screw seats placed along gaps—for fixing Velcro straps (Fig. 20 (d)). However, long-term usage and limb swelling may cause tissue herniation into the lattice structure. To preventing such occurrences, holes within the lattice structure must be shrunk by adjusting relevant parameters or hole distribution in the concerned area must be altered. Besides, use of a flexible gauze to encapsulate the affected limb, prior to wearing the splint, must be considered as part of the immobilization treatment. Through use of the above methods, user comfort can be ensured during fitting and rounded splint edges can be created to effectively buffer the friction between the splint and limb skin; nonetheless, medical-grade filaments that satisfy ISO-10993-10 standards (tests for irritation and skin sensitization), such as the Fabrial-R (JSR) material, must be employed in future splint fabrication to ensure biocapability.

Although FEA simulations confirm attainment of the minimum strength required during splint modeling, and no critical discomfort was detected during fit tests, fracture recovery usually takes several weeks to months. A long-term splint-wearing experiment must, therefore, be performed to accurately assess material stability and changes of patient comfort. Such experiments are intended to be performed as part of further research. Patient skin perspiration, limb compression, and occurrence of minor accidents during treatment may cause the splint material to age or wear and even partially break. In view of these, regular follow-up and replacement of splint parts on a monthly or biweekly is highly recommended.

### Design openness and extended applications

**Fig. 21:**
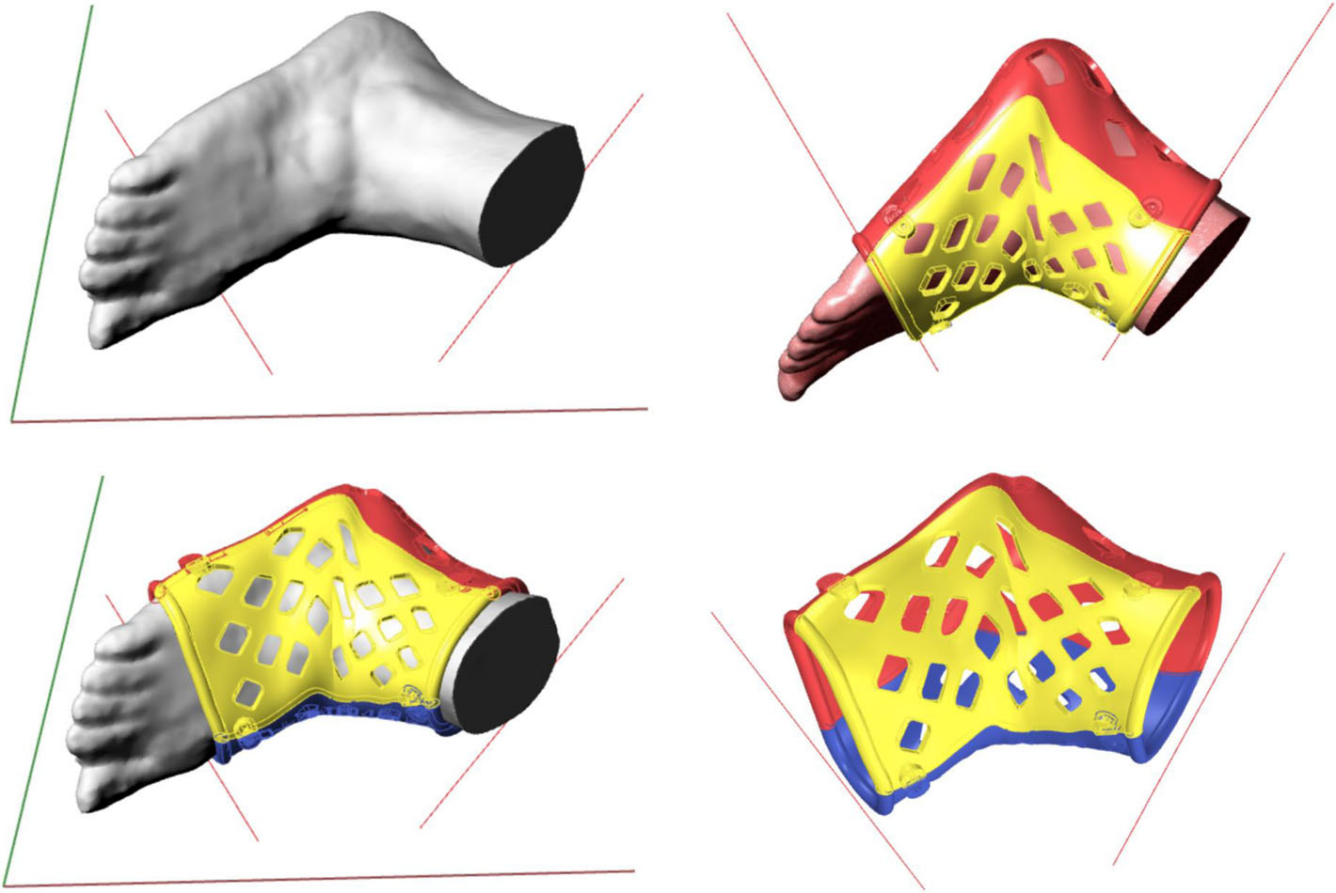
Wearable devices generated based on anatomic models of feet

The customization system employed in this study is not based on a secure software, but rather on an approximately 200-kb binary Grasshopper file that is workable in the suggested software. The system, therefore, is vulnerable and easy to disseminate and/or modify. Besides the upper limb, the proposed program is equally applicable to anatomic models of other body parts, such as the trunk and lower limb, to generate wearable devices, as depicted Fig. 21. However, the screw-system standard and calculations concerning the thickness and lattice structure need to be reinforced and tested carefully to ensure the necessary strength and safety. Any CAD designer or engineer familiar with programming tools can maintain the system with a Creative Commons Attribution license and develop extended versions based on the Grasshopper file to assist other treatment purposes. However, Rhinoceros 3D is not open source, and its commercial, educational, or trial license is required for exporting STL files of splint models. The study identifies a new collaborative relationship between the system designer and clinician to mold their own splint customization processes by exchanging their knowledge in medicine, design engineering, and 3D printing. The concept of the customization system as an open source can assist in the distribution of splint-parameter big data based on accumulated individual practical experiences.

## Conclusion

This study proposes a programmable modeling tool for splint customization to overcome scanning- and modeling-process-related problems encountered during a digitized process. For designers and engineers interested in the development of similar systems, the study demonstrates the exact step-by-step building process and describes the necessary modeling logic, possible issues caused by scanning flaws, and corresponding solutions. A comprehensive discussion of the calculation process enables one to realize how the system determines splint thickness, lattice-structure pattern, and assembly method to response to requirements of different limbs wjilst reducing the overall process duration. The study also facilitates clinicians to accomplish splint designs within few minutes through use of the semi-automatic tool without the need for prior CAD knowledge and/or post-production skills. Although the proposed method reduces the duration of 3D-scanning, CAD manipulation, and printing stages to a few hours, the total duration of the design process still exceeds that of transitional splinting, which can be accomplished within 20 min. Therefore, design-development and generation of simple prefabricated splints must be considered for providing immediate and temporary immobilization before 3D-printed splints could be made available.
